# Transcriptomic and metabolomic analysis clarify the molecular mechanisms underlying the formation of sexual and apomictic Persian walnut (*Juglans regia* L.) embryos

**DOI:** 10.3389/fpls.2025.1567247

**Published:** 2025-05-01

**Authors:** Chunjie Bao, Hong Chen, Haoliang Zhou, Feng Chen

**Affiliations:** ^1^ College of Forestry and Landscape Architecture, Xinjiang Agricultural University, Urumqi, Xinjiang, China; ^2^ Key Laboratory of Forestry Ecology and Industrial Technology in Arid Area, Xinjiang Education Department, Urumqi, Xinjiang, China

**Keywords:** Persian walnut, *Juglans regia*, apomixis, embryo, transcriptome, metabolome

## Abstract

**Purpose:**

Persian walnut (*Juglans regia* L.) is one of the world’s economically significant dry fruits, which stems from the high nutritional value of its kernel and its uses in diverse industries. Walnuts species can employ sexual and apomictic reproductive strategies. Multi-omics analyses of apomictic walnut embryos have not yet been conducted. This study integrates transcriptomic and metabolomic analyses to reveal new insights into the formation of sexual and apomictic walnut embryos, providing a valuable foundation for future research on apomictic embryo development in walnuts.

**Method:**

To elucidate the mechanisms underlying these reproductive modes, transcriptomic and metabolomic analyses were performed on the embryos of sexual and apomictic walnut species at different developmental stages.

**Results:**

Our findings revealed 321 differentially expressed genes (DEGs) and 19 differentially accumulated metabolites (DAMs) in apomictic *vs*. sexual walnut embryos. The joint transcriptomic and metabolomic analysis revealed that DEGs and DAMs were mainly enriched in metabolic pathways, biosynthesis of secondary metabolites, plant hormone signal transduction, and tryptophan metabolic pathways. The content of DAMs, such as tryptamine, jasmonic acid (JA), and JA-isoleucine, was significantly higher in embryos derived from flowers that had been forced to reproduce apomictically (subjected to polyvinyl alcohol-capped stigma treatment) than embryos derived from flowers that had been subjected to normal artificial pollination. *COMT*, *PME*, *TAT*, *CHIB*, *FG3*, *CYP82C4*, *CYP82G1*, *aceB*, *SDR*, *ribBA*, *AFS1*, *BHMT2*, *GN1_2_3*, *SGR*, *BAK1*, *trpB*, *AOC3*, *ASN*, *IAA*, *TDC*, *ZEP*, *JAZ*, and *ACO* were positively correlated with DAMs. 9 genes related to DAMs were verified by real-time quantitative PCR, and their relative expression differences were consistent with the results of the transcriptome analysis. *BAK1*, *trpB*, *AOC3*, *ASN*, *IAA*, *TDC*, *ZEP*, *JAZ*, *ALDH*, and *ACO* played a role in regulating the formation of apomictic embryos in walnut by regulating DAMs, such as auxin(tryptamine) and JA.

**Conclusion:**

TRA, JA, and JA-ILE play important roles with metabolites involved in apomixis. BAK1, trpB, AOC3, ASN, IAA, TDC, ZEP, JAZ, ALDH, and ACO may be the key genes involved in apomixis. These candidate genes could be strongly associated with the molecular mechanisms underlying apomixis in walnut were identified, and this will help clarify the formation of apomictic embryos in walnut.

## 1 Introduction

Apomixis is a type of asexual reproduction in which fertilization does not occur; consequently, the maternal haploid gamete gives rise to the embryo and asexual seeds (Hojsgaard et al., 2014). Asexual seed production has been documented in less than 1% of angiosperms (Asker and Jerling, 1992). Various alterations in the developmental program during the formation of the female germline underlie differences between apomixis and sexual reproduction. Mutations that destabilize meiosis (megasporogenesis), the gametophyte (embryo sac), and egg formation distinguish different types of apomixis. Apomixis can be classified into two types, gametophytic and sporophytic apomixis (Asker and Jerling, 1992; Nogler, 1984). In apomixis, offspring are produced directly by the female parent, and offspring have the same genetic material as the female parent and thus retain the female parent’s traits.

Apomixis has high utility for the breeding of agricultural crops and seed production (Fei, 2022; Chen et al., 2019b), as it can facilitate the fixation of heterosis, reduce breeding costs, and shorten breeding cycles (Hu et al., 2022a). The genetic control of apomixis in gametophytic apomicts is mediated by various mechanisms associated with the regulation of gene expression, including protein degradation, transcription, cell cycle control, stress responses, hormonal pathways, cell-to-cell signaling, and epigenetic mechanisms (Hand and Koltunow, 2014; León-Martínez and Vielle-Calzada, 2019; Schmidt, 2020). The identification of several common genes that are differentially expressed in multiple stages of apomixis and sexual seed production in recent studies has suggested that sexual reproduction and apomictic reproduction involve closely related developmental pathways (Brukhin, 2017). Mutations of evolutionarily conserved type II topoisomerase (*SPO11-1*), meiotic recombination protein (*REC8*), and MYB family transcription factor (*OSD1*) genes underlie apomixis in *Arabidopsis thaliana* (Marimuthu et al., 2011). Transcriptomic studies of *Poa pratensis* have shown that regulation of the expression of homeodomain transcription factor (*WUS*) and *AGO* protein (*AGO*) genes in nucellar cells or regulation of the expression of auxin-related genes, auxin 2 (*AUX2*) and auxin 3 (*AUX3*), the cytokinin-related gene cytokinin oxidase/dehydrogenase 1 (*CKX1*), and isopentenyl transferase 8 (*IPT8*) can result in the generation of apomictic embryos in *Poa pratensis* (Zhang, 2023). Khanday et al. (2019) found that the baby boom (BABY BOOM1, *BBM1*) gene can induce the apomixis of rice (*Oryza sativa*) eggs using CRISPR/Cas-9 technology, and this can facilitate the construction of an apomictic system in rice.

The formation of apomictic embryos also requires the synergistic interplay of multiple plant hormones. Some research has shown that elevated levels of auxin (IAA) (Wang, 2013; Li et al., 2022; Siena et al., 2022) and gibberellin (GA) (Park et al., 2017; Ferreira et al., 2018) enhance the development of unfertilized embryo sacs and trigger seed formation. Activating the cytokinin (CK) signaling pathway can further promote apomictic embryo formation (Zhang et al., 2024). Some studies suggest that ABA maintains embryonic cell viability and promotes embryo expansion, thereby regulating apomictic seed formation although the high levels of abscisic acid (ABA) may inhibit embryo development (Chin-Atkins et al., 1996; Fei et al., 2021). Additionally, it promotes embryo development through cell elongation, leading to embryo expansion.The reduction in zeatin (ZT) content is closely associated with embryo abortion (Gusakovskaya & Blintsov 2004).

Walnut (*Juglans regia*) is a perennial deciduous fruit tree in the family Juglandaceae. It is one of the world’s four most economically significant dry fruits, which stems from the high nutritional value of its fruit and its uses in diverse industries (Wu, 1984; Xi and Zhang, 1992). It is one of the most economically significant fruit trees associated with the development of the forest and fruit industry in Xinjiang, China (Chen and Li, 2023; Wang et al., 2013). Previous studies have shown that walnuts can reproduce via gametophytic apomixis, in which the embryo sac originates from the megaspore mother cell either directly by mitosis and/or after interrupting meiosis. Apospory occurs when a somatic, unreduced cell of the nucellus develops into an embryo sac (Liu and Sun, 2010; Zhang et al., 2014; Xiong et al., 2019) Walnut can reproduce via apomixis, and the apomixis rate of different walnut varieties ranges from 2.77% to 44.89% (Zhang et al., 2014; Xiong et al., 2019). ‘Xinxin 2’ is the main cultivar of walnut used in the development of forests and the fruit industry in the southern Xinjiang Basin. It has a thin skin, and its fruit is highly popular among consumers for its taste; in addition, its nucleolus is complete, the kernel can be easily extracted, and its kernel rate is high (Shang et al., 2018).

Preliminary investigations of the walnut research group of the College of Forestry and Landscape Architecture of Xinjiang Agricultural University have revealed that the apomixis rate of walnut can be as high as 58% (data not shown). Research on walnut apomixis has mainly focused on embryonic morphology (Chen et al., 2000; Wu et al., 2010; Ning et al., 2014; Aysen et al., 2019), fruit growth and development (Cosmulescu et al., 2012; Zhang et al., 2014; Xu et al., 2017), and the genetic identification of offspring (Sun et al., 2023; Liu et al., 2011). To the best of our knowledge, this is the first study that examines the formation of walnut apomictic embryos via multi-omics analysis. Based on the results of previous studies, growth regulator substances have been shown to be involved in regulating the development of apomictic embryos of precocious walnut, and some growth regulator substances have been shown to play a major role in this process (Chen et al., 2025).

Here, we performed transcriptomic and metabolomic analyses of apomictic and sexual walnut embryos to identify differentially expressed genes (DEGs), differentially accumulated metabolites (DAMs), and the relationships between DEGs and DAMs related to apomixis. We also analyzed the functions of these DEGs and DAMs and performed metabolic pathway enrichment analysis. Quantitative real-time PCR (qRT-PCR) and other methods were used to verify candidate genes to facilitate the development and cultivation of new walnut varieties.

## 2 Materials and methods

### 2.1 Test materials and treatment

In late April 2024 (April 25, 2024), the experiment was performed at Hongqipo Farm in Aksu City, Xinjiang Uygur Autonomous Region, China. The ‘Xinxin 2’walnut variety was used in experiments; the age of the trees ranged from 14 to 15 years; there was 5 m of spacing between plants and 6 m of spacing between rows, and the trees were robust. When the stigmas of female flowers of walnut were exposed, 4,000 well-developed female flowers are selected and divided equally into two groups. 2000 of these female flowers were subjected to polyvinyl alcohol-capped stigma (CL) treatment, while the remaining 2,000 female flowers undergo normal artificial pollination with Wen 185 (CK treatment). The samples were collected at the early stage after pollination (S1), mononuclear embryo sac stage (S2), eight nuclear embryo sac stage (S3), and heart-shaped embryo stage (S4). The samples were frozen in liquid nitrogen and stored at -80°C until subsequent transcriptomic and metabolomics analyses. Three biological replicates were performed for each treatment.

### 2.2 RNA extraction, library construction, and sequencing

The RNA of walnut embryo was extracted via the ethanol precipitation method and cetyl trimethylammonium bromide method (Gong et al., 2021; Ma et al., 2023). The quality of RNA samples was determined using agarose gel electrophoresis and an ultra-micro UV-visible spectrophotometer (German Inprin Company). After the quality of the samples was determined, they were sent to Maiwei Company (Wuhan) to construct cDNA libraries; the Illumina HiSeq 4000 high-throughput sequencing platform (Inmena, USA) was used for sequencing. After the library inspection was qualified, the different libraries were pooled according to the effective concentration and the target offline data volume for Illumina paried-end sequencing, and 150 bp paired end readings were generated.

### 2.3 Transcriptome analysis

Data quality control was performed using fastp (Chen et al., 2018) to remove reads with adapters. Paired reads were removed under the following conditions, when the the number of N in any sequencing read exceeded 10% of the length of that read, and when any sequencing read contained low-quality bases (Q ≤ 20) exceeding 50% of the length of that read. Subsequent analyses were based on clean reads. After high-quality valid sequencing data (clean reads) were obtained, the index was constructed using HISAT (Kim et al., 2015), and the number of transcriptomes after reassembly and filtering was determined to obtain sequence clusters (unigenes). The unigenes were aligned to the NR (ftp://ftp.ncbi.nih.gov/blast/db), Swiss-Prot (http://www.expasy.ch/sprot), KEGG (https://www.genome.jp/kegg/), KOG (ftp://ftp.ncbi.nih.gov), and other databases to obtain protein sequences with high similarity, and annotation information for the unigenes was obtained. Fragments Per Kilobase of transcript per Million fragments mapped (FPKM) values were calculated using featureCounts for gene alignment. The FPKM method was used to estimate gene expression levels, and DESeq2 was used to analyze DEGs for the apomictic walnut embryos *vs*. normal pollinated walnut embryos. The Benjamini & Hochberg method was used to correct the p-values, and DEGs were identified using the following criteria: Padj < 0.05 and |log_2_(fold change)| ≥ 1. Gene Ontology (GO) analysis and Kyoto Encyclopedia of Genes and Genomes (KEGG) pathway enrichment analysis were performed on the DEGs.

### 2.4 Metabolite extraction and detection

The walnut metabolites were extracted following the method of Li et al. (2016) with slight modifications. Specifically, walnut embryos were ground into powder using a grinder; 50 mg of powder, 10 μL of internal standard solution, and 1 mL of extractant were mixed. The samples were vortexed for 10 min and centrifuged at 4°C and 12,000 r · min^-1^ for 5 min. The supernatant was redissolved in 80% methanol and filtered for GC-MS analysis. The liquid-phase conditions were as follows, C18 chromatographic column (1.8 μm, 100 mm × 2.1 mm, SCIEX, USA); liquid phase, A: ultrapure water, B: acetonitrile (0.04% acetic acid was added to AB); elution, 0–1.0 min A:B = 95:5, 8.0–9.0 min A:B = 5:95, and 9.1–12.0 min A:B = 95:5; and the flow rate was 0.35 mL · min^-1^. The column temperature was 40°C, and the injection volume was 2 μL in the metabolite analysis. The mass spectrometry conditions were as follows, source temperature 550°C; positive voltage, 5,500 V; and negative voltage, -4,500 V. The pressure of the air curtain gas was 35 psi.

### 2.5 Metabolomics analysis

The software Analyst 1.6.3 was used to process the data. Sample correlation analysis and univariate analysis were performed; fold change ≥ 2 and fold change ≤ 0.5 were used as the screening criteria, and KEGG functional annotation and enrichment analysis was performed.

### 2.6 Combined omics analysis

Enriched KEGG pathways for DEGs and DAMs were obtained via KEGG enrichment analysis, and metabolites with similar functions were clustered using K-means clustering analysis. Finally, genes and metabolites with |*r*| > 0.90 and *P* < 0.05 in the KEGG pathway enrichment and K-means clustering analysis were selected. Cytoscape was used to make a correlation network diagram to further analyze correlations between genes and metabolites. The dark pink triangle represents metabolites, and the pink square represents genes. The solid line indicates positive correlation, and the dotted line indicates negative correlation.

### 2.7 qRT-PCR analysis

The expression patterns of 9 key DEGs involved in metabolic pathways were screened using qRT-PCR following the method described by Zhang (2023). The primer sequences are shown in **Supplementary Table S1**. cDNA was synthesized via reverse-transcription using the MonScript ™ RTIII All-in-One Mix with dsDNase kit (Shanghai Nuoning Biotechnology Co., Ltd.), and the obtained cDNA was stored at -20°C. A QIAGEN kit (Shanghai Haoran Biotechnology Co., Ltd.) was used to perform qRT-PCR on an ABI7500 fluorescence quantitative PCR instrument (Shanghai Aiyan Biotechnology Co., Ltd.), and the thermal cycling parameters were based on those provided in the manufacturer’s instructions. *TUA* was used as the internal reference gene (5′-ATCAGTGGCAAGGAGGATGC-3′; 5′-GAGGCCAGTGCAGTTGTCAG-3′), and relative gene expression levels were calculated using the 2^-ΔΔCt^ method (Livak and Schmittgen, 2001).

### 2.8 Data processing and analysis

All experimental data were analyzed using SPSS 21.0 (https://spss.mairuan.com/buy.html) software and graphs were drawn using Origin 2024 (https://www.originlab.com/), represented by mean values ± standard deviation. Independent sample *t*-test were used to analyze the hormone content of CL and CK and the relative expression of genes.

## 3 Results

### 3.1 Variation in the content of endogenous hormones during the development of apomictic walnut embryos

Measurements of the content of endogenous hormones in apomictic and normal pollination walnut embryos at four stages were made to determine the effect of endogenous hormones on the development of apomictic walnut embryos. The content of different endogenous hormones in apomictic and normal pollination walnut embryos varied among the four stages (**Supplementary Figure S1**). At S1, the auxin content was significantly higher in apomictic walnut embryos than in normal pollination walnut embryos (*p* < 0.01). At S2 and S3, the auxin content was significantly higher in apomictic walnut embryos than in normal pollination walnut embryos (*p* < 0.001). There was no difference in the auxin content between apomictic and normal pollination walnut embryos at S4. At S1, the content of jasmonic acid was significantly higher in apomictic walnut embryos than in normal pollination embryos (*p* < 0.001). At S2, the content of jasmonic acid was significantly higher in apomictic walnut embryos than in normal pollination walnut embryos (*p* < 0.01).

### 3.2 RNA sequencing data analysis

Illumina RNA-seq analysis was performed on samples from apomictic and normal pollination walnut embryos at each of the four developmental stages; there were three biological replicates for each sample. A total of 24 cDNA libraries were obtained via transcriptome sequencing. A total of 245.01 Gb clean data were obtained via cDNA library sequencing, and the clean data of each sample reached 8 Gb. The error rates for all samples were 0.01%, the percentage of Q20 bases was greater than 98%, the percentage of Q30 bases was greater than 96%, and the GC content was greater than 43% (**Supplementary Table S2**).

### 3.3 Identification of DEGs

**Figure 1:**
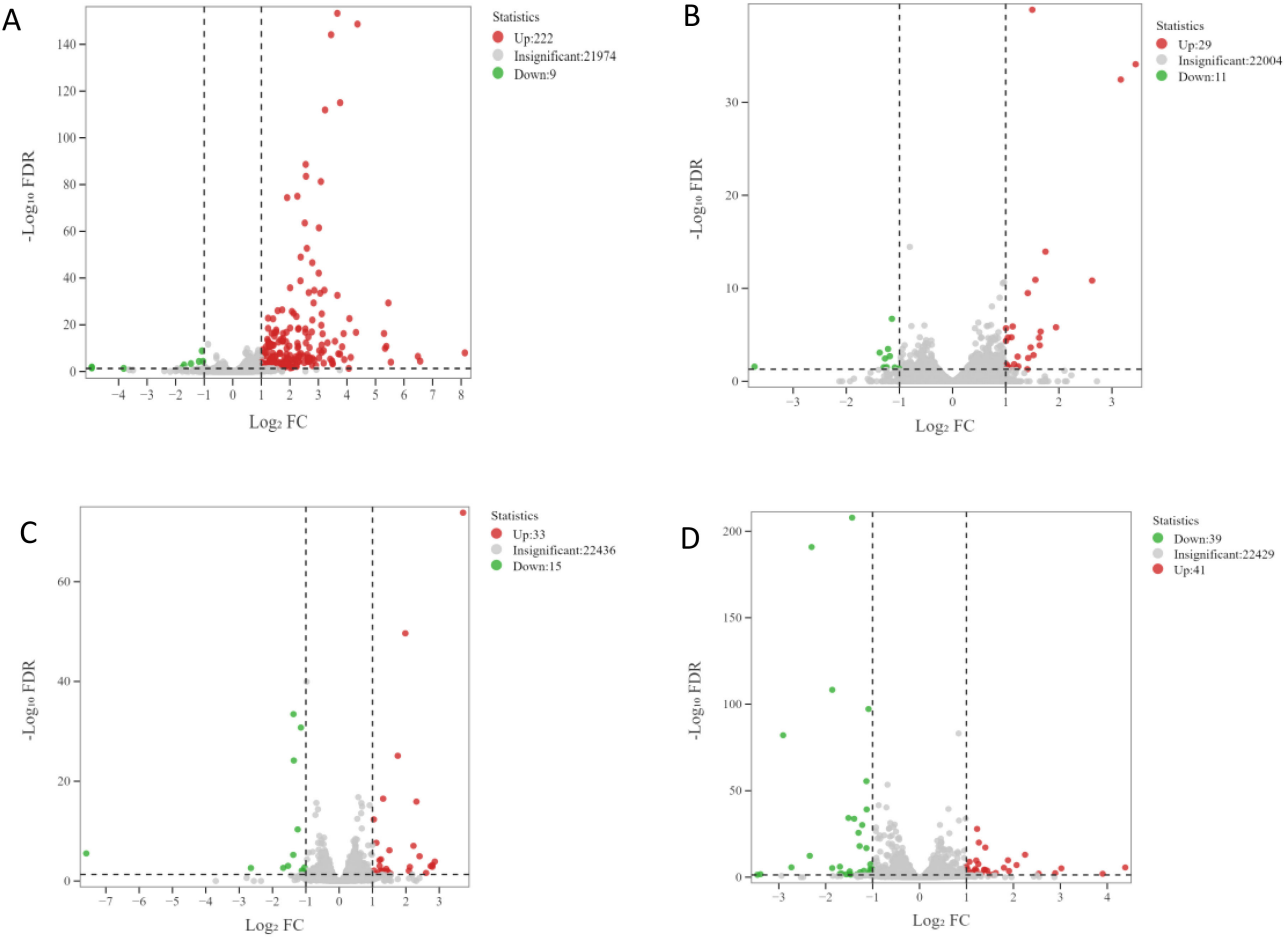
Volcano maps of DEGs. The abscissa represents the change of gene expression multiples, and the ordinate represents the significance level of differential genes. The red dots represent up-regulated differential genes, the green dots represent down-regulated differential genes, and the gray dots represent non-differentially expressed genes. **(A)** S1CL_*vs*_S1CK. **(B)** S2CL_*vs*_S2CK. **(C)** S3CL_ *vs*_S3CK. **(D)** S4CL_*vs*_S4CK. S1CL_*vs*_S1CK means the comparison between the apomictic treatment (CL) and normal pollination treatment (CK) in the stage the early stage after pollination 1 (S1) S2CL_*vs*_S2CK means the comparison between the apomictic treatment (CL) and normal pollination treatment (CK) in the mononuclear embryo sac (S2). S3CL_*vs*_S3CK means the comparison between the apomictic treatment (CL) and normal pollination treatment (CK) in the eight nuclear embryo sac stage (S3). S4CL_*vs*_S4CK means the comparison between the apomictic treatment (CL) and normal pollination treatment (CK) in the heart-shaped embryo stage (S4).

The transcriptome data were collected over four developmental stages (S1, S2, S3, and S4) in apomictic (CL) and normal pollination embryos (CK). The DEGs between the CL and CK from the same developmental stages were analyzed. A total of 321 DEGs were identified using the following criteria, log_2_(Fold Change)| ≥ 1 and false discovery rate < 0.05. The number of up-regulated and down-regulated DEGs in each group is shown in **Figure 1**. The largest number of DEGs (231) were identified in the S1CL_*vs*_S1CK group, and this included 222 up-regulated DEGs and 9 down-regulated DEGs (**Figure 1A**). The lowest number of DEGs (40) was identified in the S2CL_*vs*_S2CK group, and this included 29 up-regulated and 11 down-regulated DEGs (**Figure 1B**). In S3CL_*vs*_S3CK group, there were 33 up-regulated and 15 down-regulated DEGs (**Figure 1C**) and there are 41 up-regulated and 39 down-regulated DEGs in S4CL_*vs*_S4CK group (**Figure 1D**). An Upset diagram (**Supplementary Figure S2**) revealed that 15 DEGs were shared among the S1CL_*vs*_S1CK group and S2CL_*vs*_S2CK group. 9 DEGs were shared among the S1CL_*vs*_S1CK group and S3CL_*vs*_S3CK group. 5 common DEGs were shared among the S1CL_*vs*_S1CK group and S4CL_*vs*_S4CK group.

### 3.4 GO analysis of DEGs

GO analysis was performed, and the significance of GO terms was determined. The 321 DEGs from all samples were enriched in 104 GO terms. The number of GO terms in the biological process category was 68, the number of GO terms in the cellular component and molecular function categories was 8 and 28, respectively. Analysis of the DEGs and enriched GO terms revealed that 93.3% of up-regulated DEGs were enriched in GO terms and 62.5% of down-regulated DEGs were enriched in GO terms. Analysis of the density of DEGs revealed that 84.7% of DEGs were up-regulated and 15.3% of DEGs were down-regulated (**Supplementary Table S3**).

**Figure 2:**
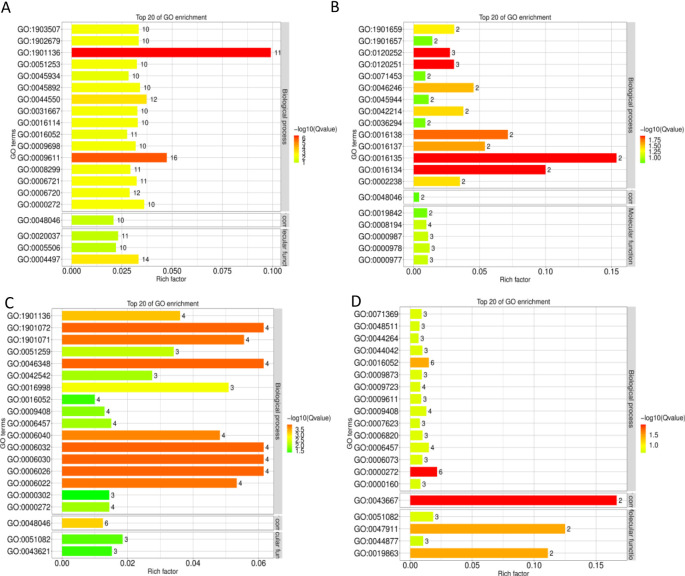
GO classification bar plots. The ordinate represents the GO entry, and the abscissa represents the ratio of the number of differential genes annotated to the entry to the total number of genes annotated to the entry. Color represents the degree of enrichment. **(A)** S1CL_*vs*_S1CK. **(B)** S2CL_*vs*_S2CK. **(C)** S3CL_*vs*_S3CK. **(D)** S4CL_*vs*_S4CK. S1CL_*vs*_S1CK means the comparison between the apomictic treatment (CL) and normal pollination treatment (CK) in the stage the early stage after pollination 1 (S1). S2CL_*vs*_S2CK means the comparison between the apomictic treatment (CL) and normal pollination treatment (CK) in the mononuclear embryo sac (S2). S3CL_*vs*_S3CK means the comparison between the apomictic treatment (CL) and normal pollination treatment (CK) in the eight nuclear embryo sac stage (S3). S4CL_*vs*_S4CK means the comparison between the apomictic treatment (CL) and normal pollination treatment (CK) in the heart-shaped embryo stage (S4).

DEGs in the S1CL_*vs*_S1CK group were significantly enriched in carbohydrate derivative catabolic process (GO: 1901136) and response to nutrient levels (GO: 0031667) (**Figure 2A**; **Supplementary Table S4**). DEGs in the S2CL_*vs*_S2CK group were significantly enriched in saponin biosynthesis process (GO: 0016135), saponin metabolism process (GO: 0016134), hydrocarbon biosynthesis process (GO: 0120251), and hydrocarbon metabolism process (GO: 0120252) (**Figure 2B**; **Supplementary Table S4**). DEGs in the S3CL_*vs*_S3CK group were significantly enriched in chitin metabolic process (GO: 0006030) and chitin catabolic process (GO: 0006032) (**Figure 2C**; **Supplementary Table S4**). DEGs in the S4CL_*vs*_S4CK group were significantly enriched in polysaccharide catabolic process (GO: 0000272) and carbohydrate catabolic process (GO: 0016052) (**Figure 2D**; **Supplementary Table S4**). DEGs were mainly enriched in GO terms related to metabolic processes, biological decomposition, and synthesis processes in the four developmental stages.

### 3.5 KEGG pathway enrichment analysis of DEGs

**Figure 3:**
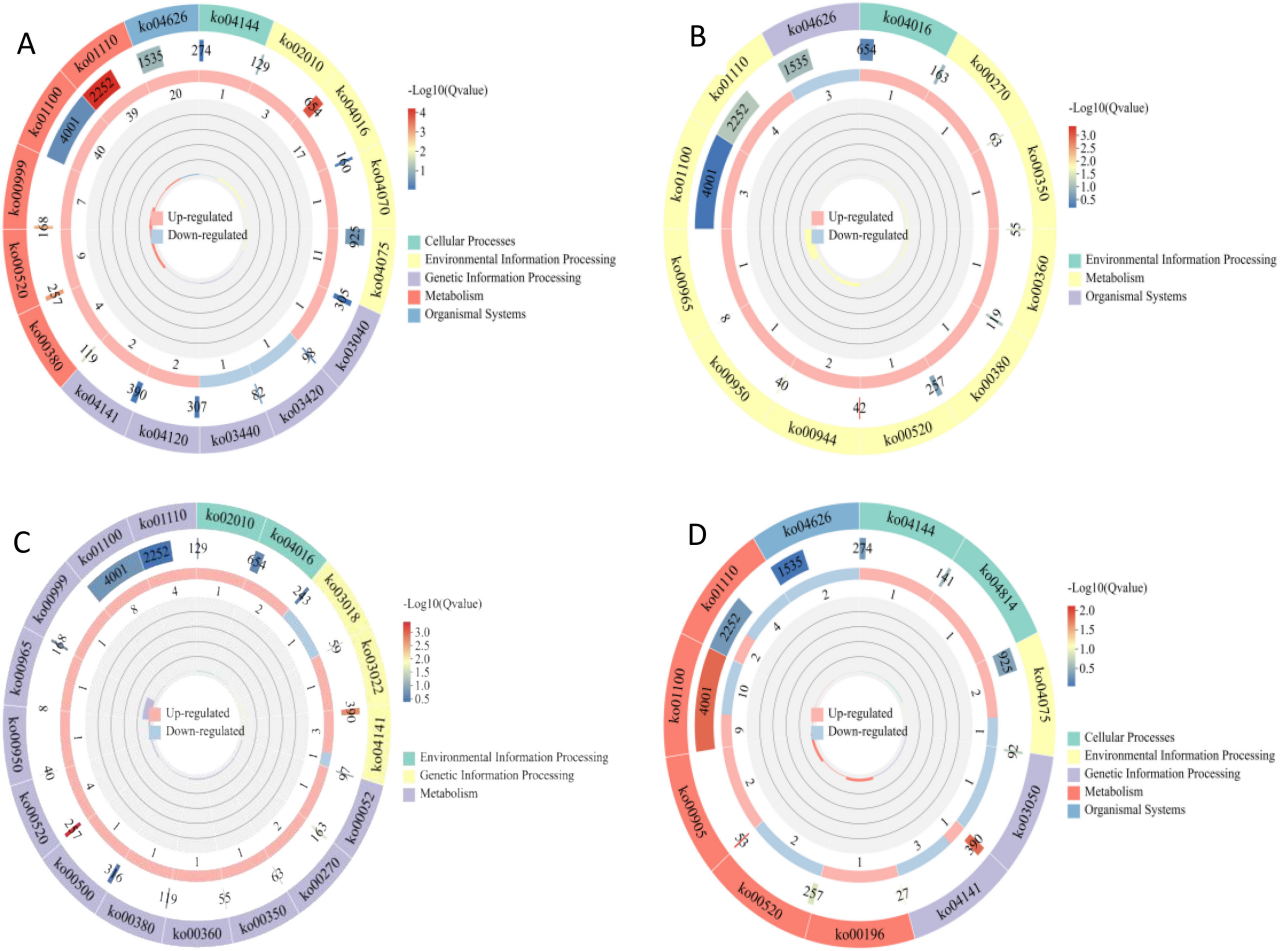
KEGG enrichment maps of DEGs. From outside to inside, the first circle is the KEGG pathway entry, and different colors represent different KEGG classifications; the second circle is the number of the classification in the background gene and q-value. Length represents the number of genes, and color represents the degree of enrichment; the third circle is up-regulated and down-regulated gene ratio bar chart, light red represents the proportion of up-regulated genes, light blue represents the proportion of down-regulated genes; the specific values are shown below; the fourth circle is the rich factor value of each category, the background auxiliary line each cell represents 0.2. **(A)** S1CL_*vs*_S1CK. **(B)** S2CL_*vs*_S2CK. **(C)** S3CL_*vs*_S3CK. **(D)** S4CL_*vs*_S4CK. S1CL_*vs*_S1CK means the comparison between the apomictic treatment (CL) and normal pollination treatment (CK) in the stage the early stage after pollination 1 (S1). S2CL_*vs*_S2CK means the comparison between the apomictic treatment (CL) and normal pollination treatment (CK) in the mononuclear embryo sac (S2). S3CL_*vs*_S3CK means the comparison between the apomictic treatment (CL) and normal pollination treatment (CK) in the eight nuclear embryo sac stage (S3). S4CL_*vs*_S4CK means the comparison between the apomictic treatment (CL) and normal pollination treatment (CK) in the heart-shaped embryo stage (S4).

In the KEGG enrichment analysis, 321 DEGs were mapped to 84 KEGG pathways, and most DEGs were enriched in metabolic process-related pathways (**Figure 3**). DEGs in the S1CK_*vs*_S1CL group were mainly enriched in metabolic pathways (ko01100), biosynthesis of secondary metabolites (ko01110), and plant hormone signal transduction (ko04075) (**Figure 3A**). DEGs in the S2CK_*vs*_S2CL group were mainly enriched in metabolic pathways (ko01100), biosynthesis of secondary metabolites (ko01110), and biosynthesis of flavonoids and flavonols (ko00944) (**Figure 3B**). The S3CK_*vs*_S3CL group was mainly enriched in metabolic pathways (ko01100), biosynthesis of secondary metabolites (ko01110), and amino sugar and nucleotide sugar metabolism (ko00520) (**Figure 3C**). DEGs in the S4CK_*vs*_S4CL group were mainly enriched in metabolic pathways (ko01100), biosynthesis of secondary metabolites (ko01110), and plant hormone signal transduction (ko04075) (**Figure 3D**).

### 3.6 Genes potentially related to apomixis

Genes that might be related to apomixis in walnut comprised DEGs that were enriched in common KEGG pathways. 88 DEGs were enriched in common KEGG pathways in the four developmental stages of apomictic and normal pollination walnut embryos (**Supplementary Table S5**). Most of the DEGs related to apomixis were present in the S1CL_*vs*_S1CK group and the S4CL_*vs*_S4CK group. 9 DEGs were expressed in at least two groups, 77 DEGs were up-regulated, and 9 DEGs were down-regulated. Most genes related to apomixis were expressed in the S1CL_*vs*_S1CK group.

### 3.7 Metabolite statistics and quality control analysis of mature walnut embryos

Metabolomics analysis revealed changes in metabolites in apomictic and normal pollination embryos in the four developmental stages. 68 metabolites were detected by liquid chromatography–tandem mass spectrometry in CL_*vs*_CK. These metabolites were divided into eight categories (**Supplementary Figure S3A**), cytokinin (CK, 21 metabolites), auxin (Auxin, 18 metabolites), gibberellin (GA, 13 metabolites), jasmonic acid (JA, 8 metabolites), salicylic acid (SA, 5 metabolites), abscisic acid (ABA, 1 metabolite), ethylene (ETH, 1 metabolite), and strigolactones (SLs, 1 metabolite). A correlation diagram of the three biological replicates of each sample (**Supplementary Figure S3B**) revealed a high degree of similarity for each comparison group, indicating that the reliability of the metabolomics data was high.

### 3.8 Identification of DAMs

**Figure 4:**
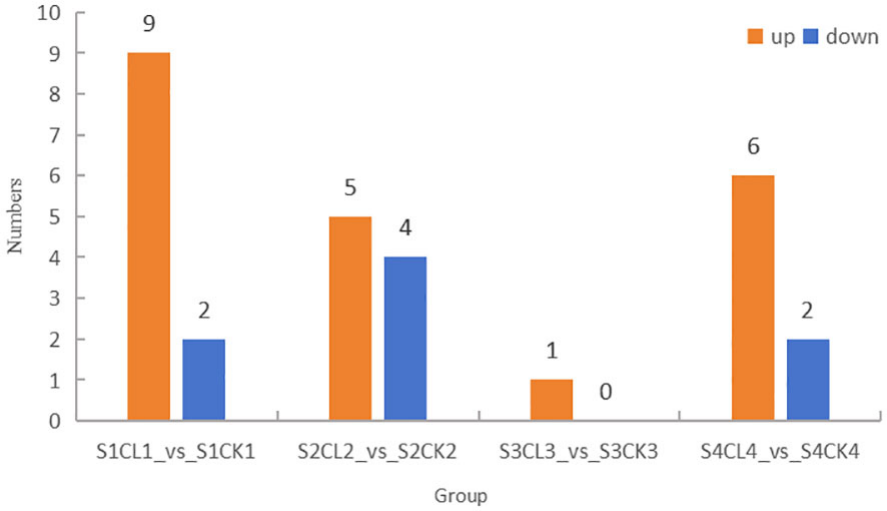
Distribution of DAMs among groups. The ordinate represents the number of differential metabolites, and the abscissa represents the group. Yellow represents up-regulated differential metabolites, and blue represents down-regulated differential metabolites. S1CL_*vs*_S1CK means the comparison between the apomictic treatment (CL) and normal pollination treatment (CK) in the stage the early stage after pollination 1 (S1). S2CL_*vs*_S2CK means the comparison between the apomictic treatment (CL) and normal pollination treatment (CK) in the mononuclear embryo sac (S2). S3CL_*vs*_S3CK means the comparison between the apomictic treatment (CL) and normal pollination treatment (CK) in the eight nuclear embryo sac stage (S3). S4CL_*vs*_S4CK means the comparison between the apomictic treatment (CL) and normal pollination treatment (CK) in the heart-shaped embryo stage (S4).

DAMs were identified using the following criteria: VIP ≥ 1 and fold-change ≥ 2 and ≤ 0.5; changes in DAMs in apomictic and normal pollination embryos in the four stages were analyzed. The greatest number of 11 DAMs was identified in the S1CL_*vs*_S1CK group, and the lowest number of 1 DAMs was identified in the S3CL_*vs*_S3CK group (**Figure 4**). A total of 19 DAMs were identified in the four groups, and this included 14 up-regulated DAMs and 5 down-regulated DAMs. The DAMs were divided into 6 categories, auxin (4 DAMs), CK (3 DAMs), GA (3 DAMs), JA (6 DAMs), SA (2 DAMs), and SL (1 DAM) (**Supplementary Table S6**).

### 3.9 KEGG pathway analysis of DAMs

**Figure 5:**
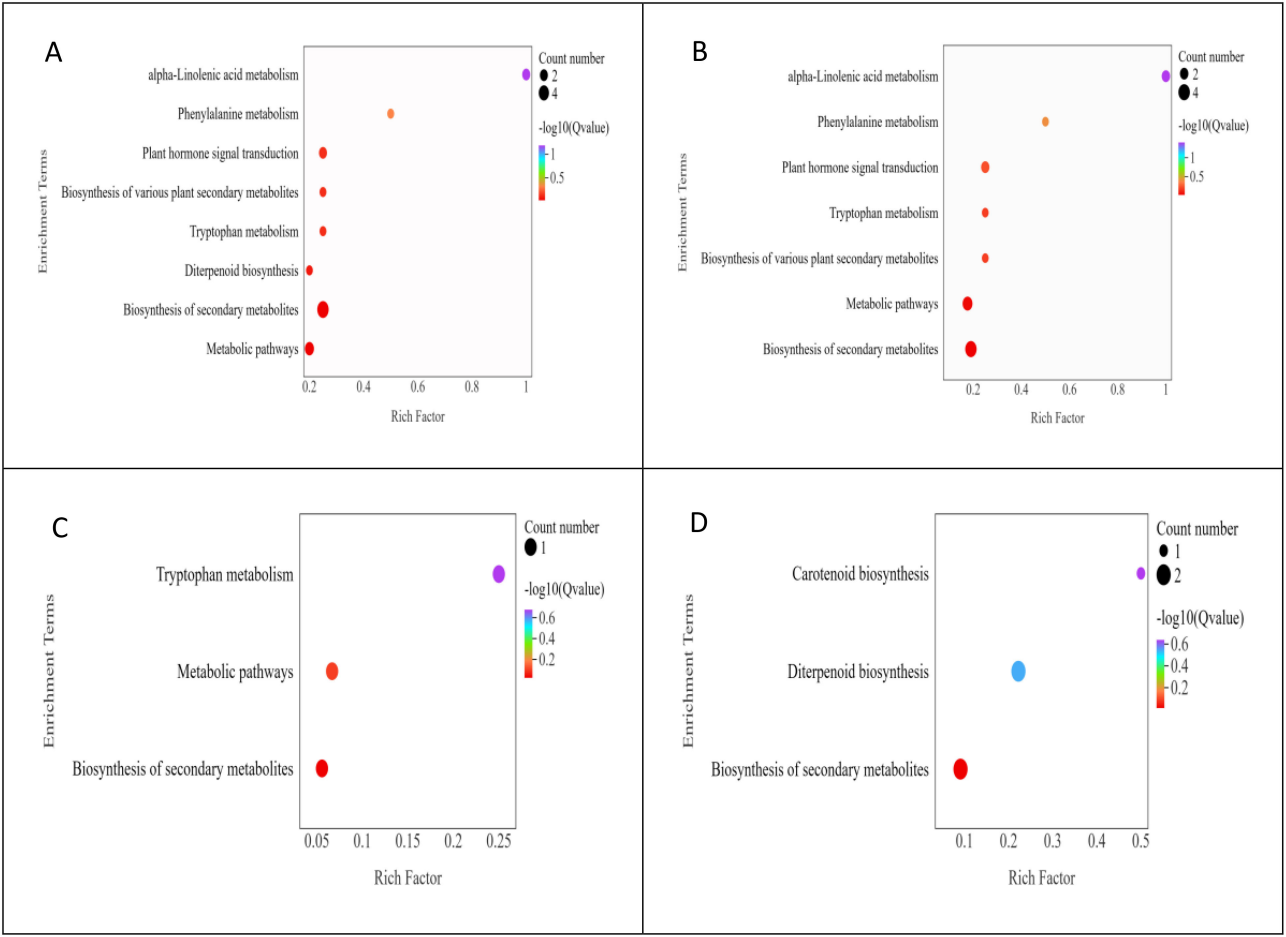
KEGG bubble diagram of DAMs. The ordinate represents the KEGG pathway, and the abscissa represents the ratio of the number of differential genes annotated to the entry to the total number of genes annotated to the entry. Color represents the degree of enrichment. The size of the point represents the number of differential genes, and the color of the point represents the degree of enrichment. **(A)** S1CL_*vs*_S1CK. **(B)** S2CL_*vs*_S2CK. **(C)** S3CL_*vs*_S3CK. **(D)** S4CL_*vs*_S4CK. S1CL_*vs*_S1CK means the comparison between the apomictic treatment (CL) and normal pollination treatment (CK) in the stage the early stage after pollination 1 (S1). S2CL_*vs*_S2CK means the comparison between the apomictic treatment (CL) and normal pollination treatment (CK) in the mononuclear embryo sac (S2). S3CL_*vs*_S3CK means the comparison between the apomictic treatment (CL) and normal pollination treatment (CK) in the eight nuclear embryo sac stage (S3). S4CL_*vs*_S4CK means the comparison between the apomictic treatment (CL) and normal pollination treatment (CK) in the heart-shaped embryo stage (S4).

The results of the KEGG pathway enrichment analysis of DAMs in apomictic and normal pollination embryos in the four developmental stages are shown in **Figure 5**. The main KEGG pathways were mainly divided into two categories, 6 metabolism and 1 environmental information processing. DAMs in the S1CK_*vs*_S1CL group were mainly enriched in metabolic pathways (ko01100), biosynthesis of secondary metabolites (ko01110), and plant hormone signal transduction (ko04075) (**Figure 5A**). DAMs in the S2CK_*vs*_S2CL group were mainly enriched in metabolic pathways (ko01100), biosynthesis of secondary metabolites (ko01110), and plant hormone signal transduction (ko04075) (**Figure 5B**). DAMs in the S3CK_*vs*_S3CL group were mainly enriched in metabolic pathways (ko01100) and biosynthesis of secondary metabolites (ko01110) (**Figure 5C**). DAMs in the S4CK_*vs*_S4CL group were mainly enriched in the biosynthesis of secondary metabolites (ko01110) and diterpenoid biosynthesis (ko00904) (**Figure 5D**).

### 3.10 Combined transcriptomic and metabolomic analysis

**Figure 6:**
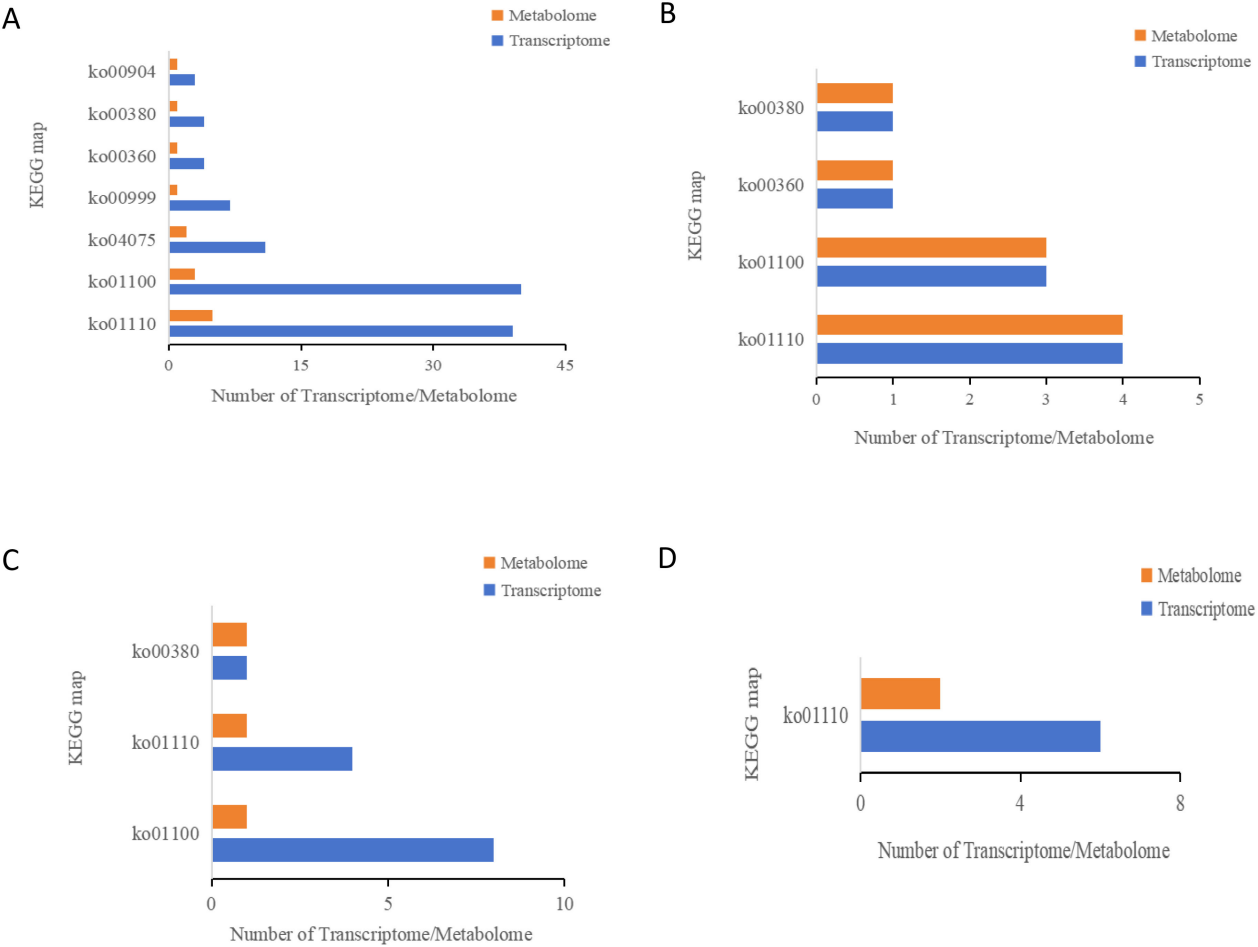
KEGG enrichment analysis bar chart. The ordinate represents the KEGG pathway, and the abscissa represents the differential genes and differential metabolites of the transcriptome and metabolites on this pathway. Yellow represents the metabolome, and blue represents the transcriptome. ko01110: Biosynthesis of secondary metabolites. ko01100: Metabolic pathways. ko04075: Plant hormone signal transduction. ko00999: Biosynthesis of various plant secondary metabolites. ko00360: Phenylalanine metabolism. ko00380: Tryptophan metabolism. ko00904: Diterpenoid biosynthesis. **(A)** S1CL_*vs*_S1CK. **(B)** S2CL_*vs*_S2CK. **(C)** S3CL_*vs*_S3CK. **(D)** S4CL_*vs*_S4CK. S1CL_*vs*_S1CK means the comparison between the apomictic treatment (CL) and normal pollination treatment (CK) in the stage the early stage after pollination 1 (S1). S2CL_*vs*_S2CK means the comparison between the apomictic treatment (CL) and normal pollination treatment (CK) in the mononuclear embryo sac (S2). S3CL_*vs*_S3CK means the comparison between the apomictic treatment (CL) and normal pollination treatment (CK) in the eight nuclear embryo sac stage (S3). S4CL_*vs*_S4CK means the comparison between the apomictic treatment (CL) and normal pollination treatment (CK) in the heart-shaped embryo stage (S4).

An integrated transcriptomic and metabolomic analysis was conducted to clarify the regulatory mechanism underlying the development and synthesis pathways of apomictic embryos. In our study, KEGG enrichment analysis was performed on DEGs and DAMs identified using apomictic and normal pollination embryos at four developmental stages (**Figure 6**). In the S1CK_*vs*_S1CL group, 108 DEGs and 14 DAMs were enriched in seven KEGG pathways (**Figure 6A**); nine DEGs and nine DAMs in the S2CK_*vs*_S2CL group were enriched in four KEGG pathways (**Figure 6B**). In the S3CK_*vs*_S3CL group, 13 DEGs and 3 DAMs were mapped to three KEGG pathways (**Figure 6C**); six DEGs and two DAMs in the S4CK_*vs*_S4CL group were enriched in one KEGG pathway (**Figure 6D**). The results of the joint transcriptomic and metabolomic analysis revealed that these DEGs and DAMs were mainly enriched in four pathways, metabolic pathway (ko01100), biosynthesis of secondary metabolites (ko01110), plant hormone signal transduction (ko04075), and tryptophan metabolism (ko00380).

### 3.11 Correlation analysis of the metabolomic and transcriptomic data

**Figure 7:**
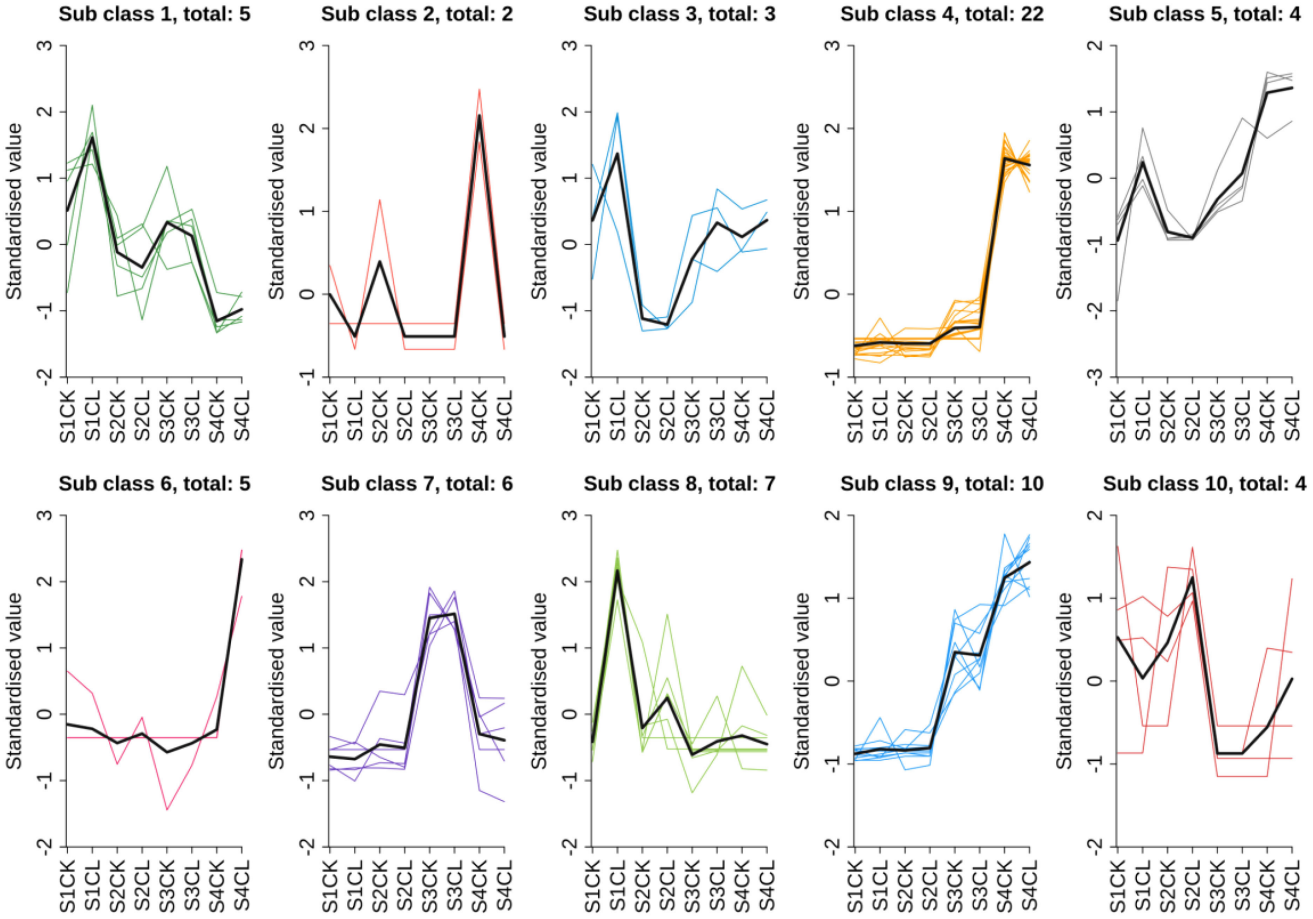
Results of the k-means clustering analysis. The ordinate represents the standardized material content, and the abscissa represents the sample grouping name. The Sub class represents the number of material classification clusters with the same change trend, and the number after total represents the number of substances in this classification cluster. S1CL means standardized value of the apomictic treatment (CL) in the stage the early stage after pollination 1 (S1). S1CK means standardized value of the normal pollination treatment (CK) in the stage the early stage after pollination 1 (S1). S2CL means standardized value of the apomictic treatment (CL) in the mononuclear embryo sac (S2). S2CK means standardized value of the normal pollination treatment (CK) in the mononuclear embryo sac (S2). S3CL means standardized value of the apomictic treatment (CL) in the eight nuclear embryo sac stage (S3). S3CK means standardized value of normal pollination treatment (CK) in the eight nuclear embryo sac stage (S3). S4CL means standardized value of apomictic treatment (CL) in the heart-shaped embryo stage(S4). S4CK means standardized value of normal pollination treatment (CK) in the heart-shaped embryo stage (S4).

To further clarify metabolic changes during the development of walnut embryos, 68 metabolites in walnut embryos were divided into 10 clusters using the K-means clustering algorithm (**Figure 7**). We identified enriched metabolites in apomictic and normal pollination embryos at four developmental stages. The content of the metabolites in the first cluster gradually decreased in the four developmental stages, and the overall trend was downward. The content of metabolites in the second cluster was higher in the normal pollination walnut embryos than in apomictic embryos; the content of metabolites in cluster 3 and 10 gradually increased during the late stage of walnut embryo formation. The content of metabolites in the 4, 5, 6, and 9 clusters gradually increased during the formation of walnut embryo, and the overall trend was upward. The content of metabolites in the 7 cluster was highest at S3. The content of metabolites in the 8th cluster was higher in apomictic embryos in the four developmental stages than in normal pollination embryos; the overall trend was upward. In general, the overall trend in the content of metabolites in the eight clusters of 1, 3, 4, 5, 6, 7, 9, and 10 in apomictic and normal pollination walnut embryos during different developmental stages was the same.

**Figure 8:**
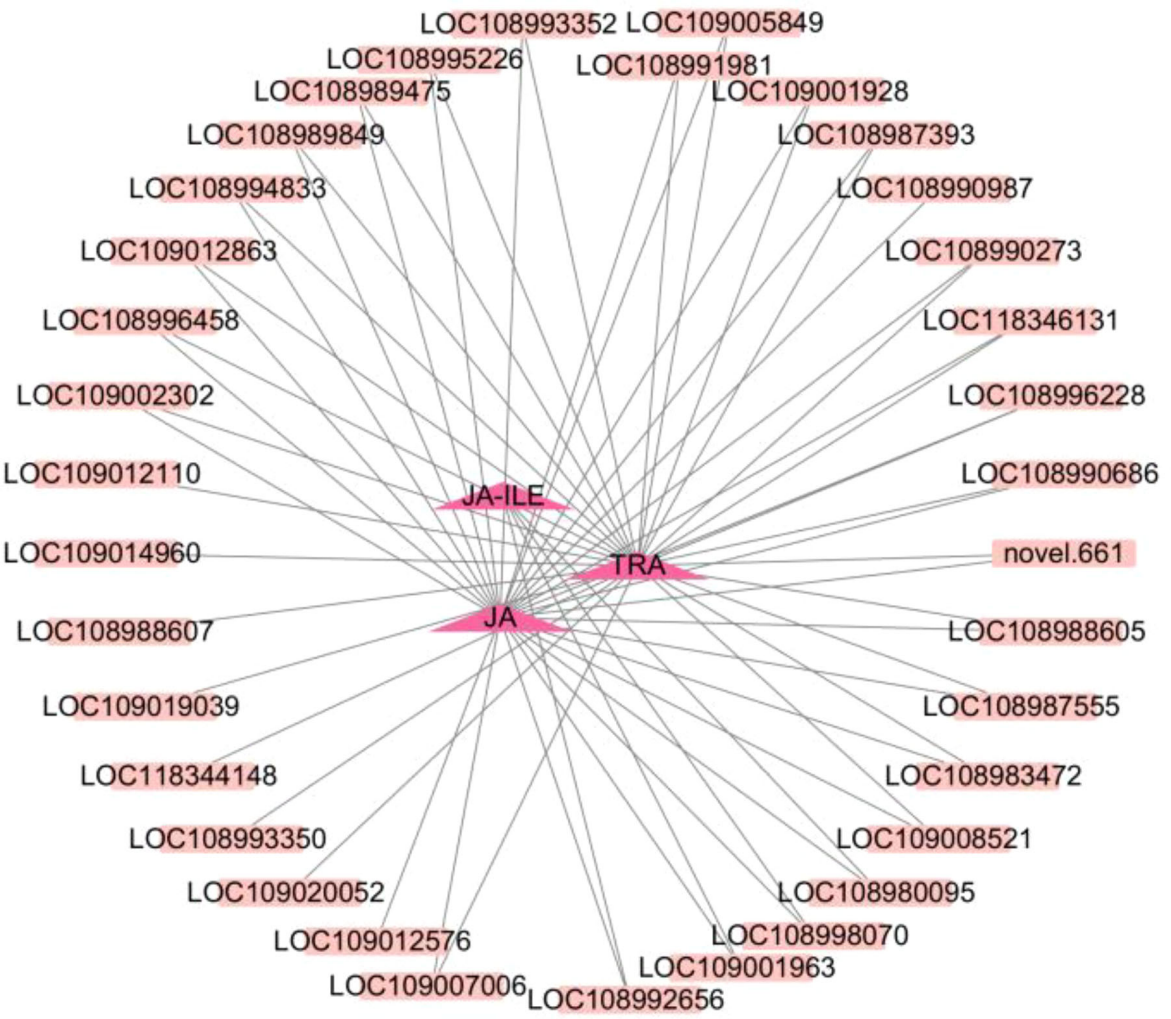
Correlation network diagram. The dark pink triangle represents metabolites, and the pink square represents genes. The solid line indicates positive correlation.

To clarify the relationships between DEGs and DAMs based on the k-means clustering data and results of the KEGG analysis, 35 genes belonging to cluster 8 were found to be enriched in metabolism, secondary metabolite synthesis, tryptophan metabolism, and plant hormone signal transduction. Correlation analysis was performed, and metabolites in cluster 8 that met the following criteria were used in analyses: |r > 0.8| and P < 0.05 with corresponding DEGs enriched in the same KEGG pathways. Through association analysis, three DAMs [tryptamine (TRA), jasmonic acid (JA), and jasmonic acid-isoleucine (JA-ILE)] and 35 DEGs related to the three DAMs were screened (**Figure 8**; **Supplementary Table S7**). The expression of TRA, JA, and JA-ILE was 3.01, 3.46, and 8.97 times higher in CL than in the CK, respectively (**Supplementary Table S8**). Three genes including *BAK1*, *IAA* and *JAZ* were positively correlated with JA-ILE, and the gene expression was significantly up-regulated. Twenty-two genes including *CHIB*, *FG3*, *ACO*, *CYP82C4*, *TAT*, *GN1 _ 2 _ 3*, *ansB* or *ASN*, *BHMT2*, *ZEP* or *ABA1*, *AOC3* or *TynA*, *aceB* or *glcB*, *trpB*, *COMT*, *PME*, *ribBA*, *DDC* or *TDC*, *SGR*, *AFS1*, *CYP82G1*, *IAA*, *BAK1* and *JAZ* were positively correlated with JA, and the gene expression was significantly up-regulated. Nineteen genes including *ALDH*, *SDR*, *CYP82G1*, *AFS1*, *SGR*, *DDC* or *TDC*, *PME*, *COMT*, *aceB* or *glcB*, *AOC3* or *TynA*, *ZEP* or *ABA1*, *BHMT2*, *ansB* or *ASN*, *GN1 _ 2 _ 3*, *TAT*, *CYP82C4*, *CHIB*, *ACO* and *FG3* were positively correlated with TRA, and gene expression was significantly up-regulated.

### 3.12 Analysis of pathways related to apomixis

**Figure 9:**
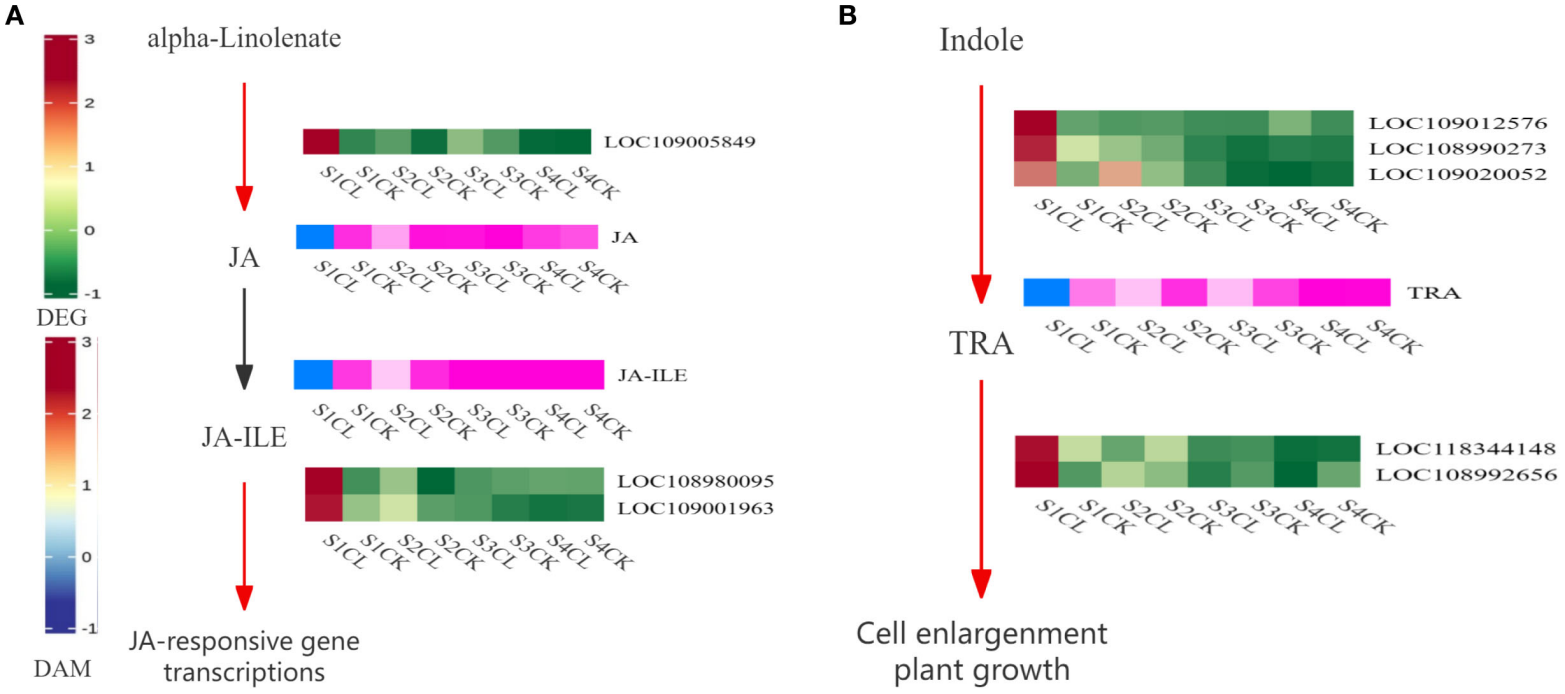
Apomixis-related pathway diagrams. **(A)** JA biosynthesis and signal transduction pathway. **(B)** Auxin biosynthesis and signal transduction pathway. Red and green indicate gene expression, and blue and pink indicate metabolite expression. Red indicates that the gene is up-regulated during this process, and green indicates that the gene is down-regulated during this process. Blue indicates that the metabolites are up-regulated during this process, and pink indicates that the metabolites are down-regulated during this process. Black indicates that genes or metabolites are not expressed during this process.

The combined transcriptomic and metabolomic analysis revealed that the development of apomictic walnut embryos is related to the plant hormone signal transduction pathway. 3 DEGs (LOC109005849, LOC108980095, and LOC109001963) and 2 DAMs (JA and JA-ILE) were related to JA biosynthesis and signal transduction (**Figure 9A**). The expression of DEGs and the content of DAMs were higher in apomictic embryos than in normal pollination embryos, and these DEGs and DAMs were both up-regulated. 5 DEGs (LOC109012576, LOC108990273, LOC109020052, LOC118344141, and LOC108992656) and 1 DAM (TRA) were related to auxin biosynthesis and signal transduction (**Figure 9B**). The expression of DEGs and the content of DAMs were higher in apomictic embryos than in normal pollination embryos.

### 3.13 qRT-PCR validation

The expression patterns of nine DEGs (*BAK1*, *JAZ, IAA, CYP82C4, COMT, ZEP/ABA1, CHIB, DDC/TDC*, and *AOC3/tynA*) were verified by qRT-PCR using *TUA* as an internal reference gene. The expression changes observed in the above 9 genes in apomictic embryos and normal pollination embryos in the four developmental stages differed (**Supplementary Figure S4**). The relative expression levels of nine genes were basically consistent with the results of the transcriptome analysis, indicating that the experimental results of this study were reliable.

## 4 Discussion

Walnut can reproduce via apomixis, and this has major implications for the fixation of heterosis in breeding programs and hybrid seed production. We used transcriptomic and apomictic and metabolomic approaches to analyze the formation of apomictic and normal pollination walnut embryos at four developmental stages. A total of 321 DEGs and 19 DAMs in the CL_*vs*_CK were obtained. Joint transcriptomic and metabolomic analysis revealed that DEGs and DAMs were mainly enriched in metabolic pathways, biosynthesis of secondary metabolites, plant hormone signal transduction, and tryptophan metabolism. The formation and development of embryos are related to the accumulation of metabolites, which lead to changes in shape and size. Cluster analysis of the metabolomic data in apomictic and normal pollination walnut embryos in four developmental stages revealed that the metabolites could be divided into 10 clusters. Correlation analysis of each cluster of metabolites and their corresponding DEGs was performed to construct a correlation network.

Plant hormones regulate various stages of plant growth and development, and the ability of nucellar cells to perform embryogenesis, one of the conditions underlying apomixis, is related to plant hormones. The main genes in apomictic walnut embryos were positively correlated with plant hormones in metabolites, indicating that walnut may carry out apomixis by regulating hormone signals through genes. JA plays an important regulatory role in plant growth and development, such as seed maturation, embryo development, leaf senescence, root elongation, and carbohydrate accumulation (FreireRios et al., 2020). The *BAK1* gene can be used as a co-receptor to form a complex with the receptor, thereby regulating the development of anther tapetum, integument development, stomatal formation, and other biological processes (Chen et al., 2019a; Li et al., 2023b; Chen and Li, 2023). The study of *SERK* family members represented by *BAK1* affects the biological processes of plant anther development and embryogenesis (Ma et al., 2016). *SERK* family genes involved in plant reproductive growth and development can lead to the early termination of sexual reproduction under certain conditions, and the whole reproductive process can be completed through apomixis (Albertini et al., 2005). In this study, *BAK1* regulated the expression of JA metabolites in apomictic embryos, which was consistent with the regulation of genes involved in apple apomixis by the *SERK* family (Zhang et al., 2015).

The high expression of JA is regulated by the *JAZ* gene, and the complex composed of the *MYC2* transcription factor and the JA signal receptor COI protein comprise the core module of the JA signal (Li et al., 2021; Zheng et al., 2023). The *JAZ* gene is a member of the plant-specific TIFY family. JAZ protein is located upstream of the JA signal transduction pathway; it affects the JA pathway and participates in the regulation of different growth and development processes of plants, such as flower (Huang et al., 2023), root growth (Han et al., 2023), fruit ripening (Hu et al., 2022b). *JAZ* is degraded by ubiquitination and releases *MYC2* to perform corresponding functions to regulate embryo development. The results are similar to those of *Arabidopsis thaliana* seeds after they enter the mature stage (Wager and Browse, 2012), indicating that the *JAZ* gene is involved in regulating apomictic embryo formation. The *AOC3* gene is an important synthetic gene involved in the JA signaling pathway in the *AOC* gene family. In this study, the expression of the *AOC3* gene was up-regulated to promote the formation of apomictic embryos, which was consistent with the detection of *cis*-acting regulatory elements involved in jasmonates associated with the expression of *AOC* family genes involved in embryo germination; this indicates that *AOC* family genes regulate embryo germination and formation (Li et al., 2020).

ABA regulates seed dormancy, germination, growth, and responses to environmental stress (Hao and Qiang, 2006). It is also involved in plant flowering (Chin-Atkins et al., 1996) and embryo development (Yan et al., 2018). *ZEP* is an ABA synthesis gene (Li et al., 2023a). In our study, up-regulation of *ZEP* promotes the development of apomictic embryos, which is consistent with the results of the development of apomictic embryo sacs in Kentucky bluegrass (Yu et al., 2024). Auxin plays an important role in the whole life cycle of plants and participates in apomixis by regulating the expression of auxin-responsive genes (Zhang, 2023; Ortiz et al., 2019). The involvement of the *ALDH* gene and *TYD* gene in the IAA pathway of tryptamine synthesis is consistent with the up-regulation of *ALDH* expression in our study. Auxin was highly expressed in apomictic embryos. The up-regulation of *IAA* and the expression of *ARF* in auxin signal transduction induce the expression of downstream auxin-induced genes, which affects the expression of *GH3* and promotes embryo development. This finding is consistent with the results of a previous study (Wang, 2013) indicating that high concentrations of *IAA* genes are involved in regulating the development of apomictic walnut embryos. The *trpB* gene and *TDC* gene play an important role in auxin synthesis and plant organ development. In our study, the up-regulation of *trpB* and *TDC* expression promotes an increase in the auxin content. When auxin reaches a certain concentration, *SAUR* responds to the auxin signal and regulates nucleolus development through auxin synthesis and transport (Peng et al., 2023; Cui et al., 2021), which is consistent with the results of this study, indicating that the *trpB* gene and *TDC* gene are also involved in regulating the development of apomictic walnut embryos.

The heading date and before and after flowering are key periods affecting apomixis in plants (Yin, 2006; Li et al., 2002). The *ACO* gene is an important rate-limiting enzyme gene involved in ETH production; *ACO* genes, which are encoded by a multi-gene family, mediate the growth and development of plants and responses to biotic and abiotic stresses by regulating the production and function of ETH in plants (Norikoshi et al., 2022). In our study, the expression of *ACO* was up-regulated during the development of apomictic walnut embryos, which was consistent with the results of studies of the development of apomictic citrus embryos (Tan et al., 2015). In our study, both the *ACO* gene and *ASN* gene were involved in the development of apomictic walnut embryos, and the results were consistent with the development of apomictic *Poa pratensis* ‘Qingshui’ embryos (Yu et al., 2024).

Genes such as *COMT, PME, TAT, CHIB, FG3, CYP82C4, CYP82G1, aceB, SDR, ribBA, AFS1, BHMT2, GN1_2_3*, and *SGR* were detected in the combined transcriptomic and metabolomic analysis, which may indirectly affect other metabolites to regulate auxin and JA and may in turn regulates the formation of apomictic embryos. However, due to the complexity of the mechanism of apomictic embryo formation, the above genes may affect the expression of auxin and JA by participating in other metabolic pathways or biosynthetic pathways. Therefore, whether the formation of apomictic embryos is related to the above genes requires confirmation by subsequent studies.

## 5 Conclusion

**Figure 10:**
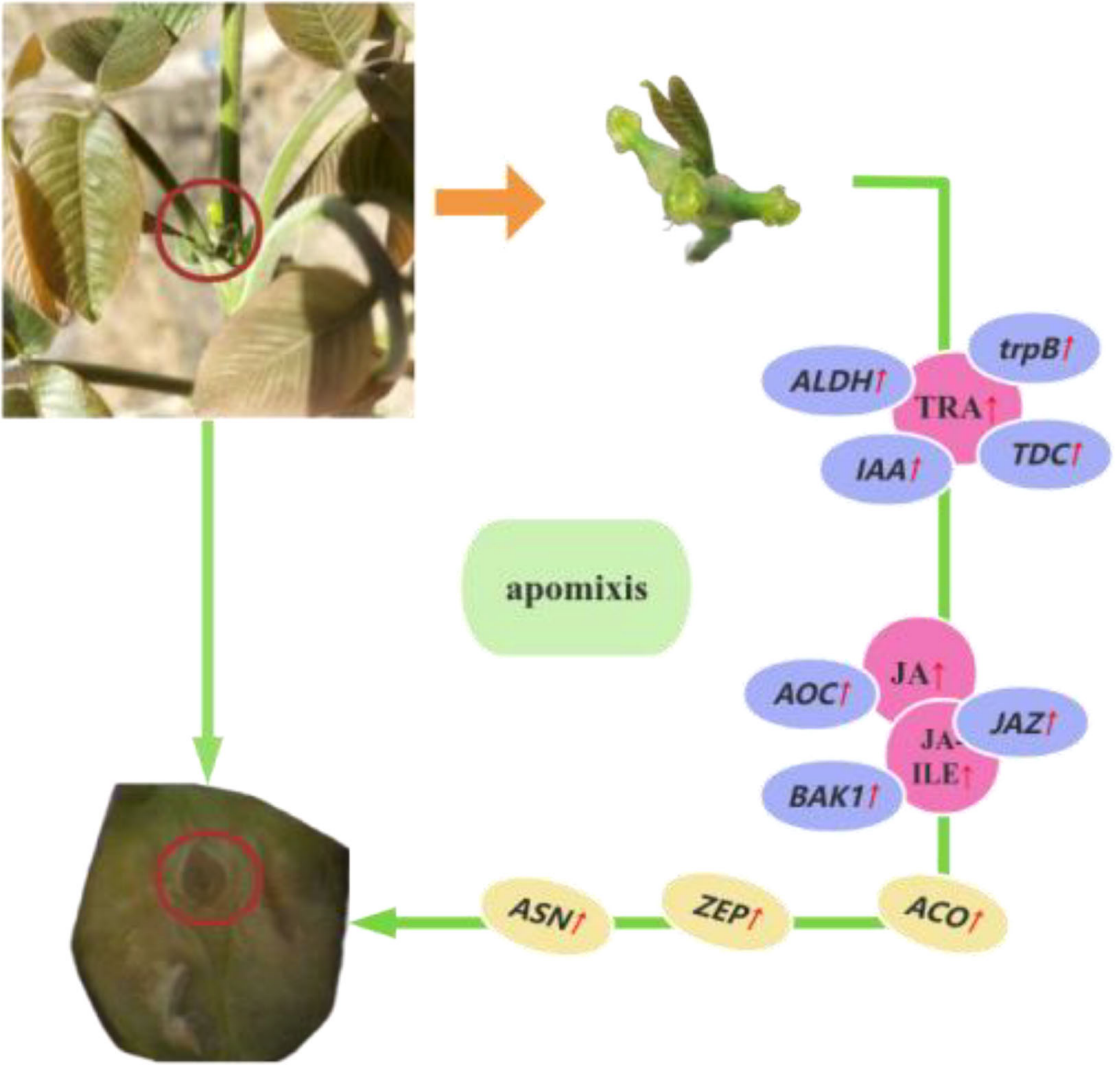
Diagram showing the regulation of apomixis-related genes and metabolites.

In our study, 321 DEGs were detected by transcriptome sequencing; 252 DEGs were up-regulated, and 69 DEGs were down-regulated. A total of 19 DAMs were identified by metabolomics; 14 DAMs were up-regulated, and 5 DAMs were down-regulated. KEGG pathway enrichment analysis and correlation network analysis revealed that TRA, JA, and JA-ILE play important roles with metabolites involved in walnut apomixis. *BAK1*, *trpB*, *AOC3*, *ASN*, *IAA*, *TDC*, *ZEP*, *JAZ*, *ALDH*, and *ACO* may be the key genes involved in walnut apomixis. Genes of *ALDH*, *IAA*, *trpB* and *TDC* positively regulate TRA production, the genes of *ALDH*, *IAA*, *trpB* and *TDC* can positively regulate the production of the TRA, the gene of *AOC* can positively regulate the production of the JA, and the *BAK1* and *JAZ* can positively regulate JA-ILE production, collectively promoting apomixis in walnuts (**Figure 10**).

## Data Availability

The data presented in the study are deposited in the Genome Sequence Archive (GSA) of China National Center for Bioinformation (CNCB), accession number "CRA024635"; https://bigd.big.ac.cn/gsa/browse/CRA024635.
