# Supplementary material for: Transcriptomic and metabolomic analysis clarify the molecular mechanisms underlying the formation of sexual and apomictic Persian walnut (Juglans regia L.) embryos
Source: Front Plant Sci. 2025 May 1;16:1567247. doi: 10.3389/fpls.2025.1567247 (PMC12078271; doi:10.3389/fpls.2025.1567247)
Supplement: Supplementary file 1 [file DataSheet1.docx]

Supplementary Material

Table S1 Primer pairs used for qRT-PCR analysis

| **Gene ID** | **Gene Name** | sequence（5'-3'） | |
| --- | --- | --- | --- |
| LOC108998070 | *BAK1* | F | TCCTGACGATGAAGTGGACG |
|  |  | R | ACGGAGCCCCAACTGAATTT |
| LOC108980095 | *JAZ* | F | ATTCACTTGGCGGTGGCATT |
|  |  | R | TAAGGGGAGGAGACGCAAGT |
| LOC108992656 | *IAA* | F | GCTTCGCCATCCACGGTAA |
|  |  | R | CGCCAACTAGCATCCAGTCA |
| LOC109014960 | *CYP82C4* | F | AAACAACGAAGCCTCTCAAGT |
|  |  | R | CCCTAAGATAAGGGTCAGGCAA |
| LOC109001928 | *COMT* | F | TCATGAGTGTCTGGGACCAT |
|  |  | R | TTTCGCGGAGTCTTCCTTCC |
| LOC108993352 | *ZEP/ABA1* | F | GGGACCATTCTCAGAACTAAGGTTT |
|  |  | R | TTATGGCAGATCTCCCTGCG |
| LOC109002302 | *CHIB* | F | ATGAAACCACAGGAGGGTGG |
|  |  | R | TGAGTTGAATGGGTCCACGG |
| LOC108990273 | *DDC/TDC* | F | GTCACATCCGACAAGAGGTTTG |
|  |  | R | TTTGTAGTACGTTTTGCGTATGCT |
| LOC109005849 | *AOC3/tynA* | F | AAGCATCAAAGTTGGGGTGGA |
|  |  | R | CGTCAACGTCAAGGTCGAGA |

| 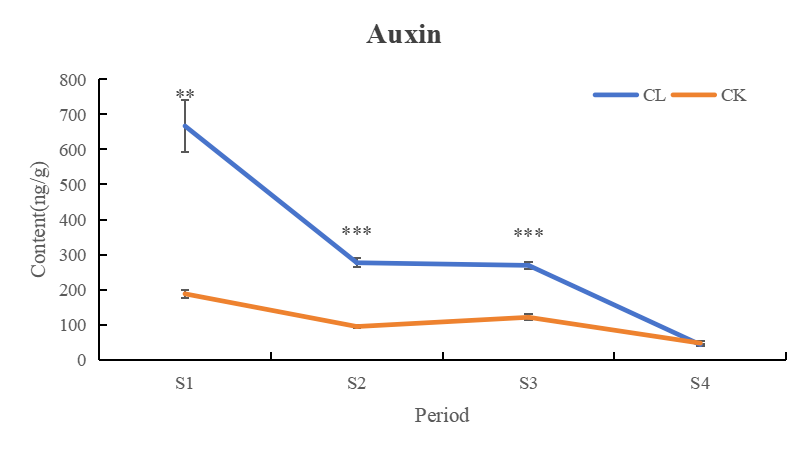  A | 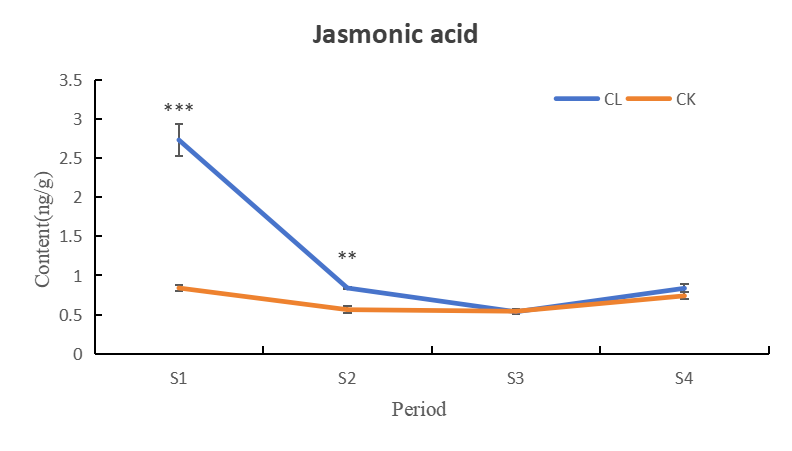  B |
| --- | --- |

Figure S1 Endogenous hormone content in walnut embryo under different treatments

Note: A: Auxin content. B: Jasmonic acid content. *, * *, and * * * indicate significant or extremely significant differences at *P* < 0.05, *P* < 0.01, and *P* < 0.001 levels, respectively.

CL means apomictic treatment. CK means normal pollination treatment. S1 means at the early stage after pollination, S2 means mononuclear embryo sac stage, S3 means eight nuclear embryo sac stage, S4 means heart-shaped embryo stage.

Table S2 Transcriptome sequencing data assembly statistics

| **Sample** | **Raw Reads** | **Clean Reads** | **Clean Base (G)** | **Error (%)** | **Q20 (%)** | **Q30 (%)** | **GC Content (%)** |
| --- | --- | --- | --- | --- | --- | --- | --- |
| S1CL-1 | 73051958 | 71734044 | 10.76 | 0.01 | 98.87 | 96.31 | 45.68 |
| S1CL-2 | 70967432 | 69957406 | 10.49 | 0.01 | 98.96 | 96.57 | 45.4 |
| S1CL-3 | 69671276 | 68677328 | 10.3 | 0.01 | 98.85 | 96.23 | 45.4 |
| S1CK-1 | 60578448 | 59788852 | 8.97 | 0.01 | 98.9 | 96.37 | 45.57 |
| S1CK-2 | 74236002 | 73316564 | 11 | 0.01 | 98.91 | 96.43 | 45.38 |
| S1CK-3 | 62880656 | 58369742 | 8.76 | 0.01 | 99.19 | 97.34 | 43.61 |
| S2CL-1 | 79371166 | 78044918 | 11.71 | 0.01 | 98.99 | 96.69 | 45.51 |
| S2CL-2 | 88457436 | 87002148 | 13.05 | 0.01 | 99.02 | 96.76 | 45.62 |
| S2CL-3 | 54528468 | 53689678 | 8.05 | 0.01 | 98.87 | 96.29 | 45.52 |
| S2CK-1 | 76193608 | 75019478 | 11.25 | 0.01 | 98.98 | 96.61 | 45.09 |
| S2CK-2 | 74899922 | 73713362 | 11.06 | 0.01 | 98.87 | 96.31 | 45.54 |
| S2CK-3 | 60319304 | 59038978 | 8.86 | 0.01 | 99.2 | 97.35 | 43.34 |
| S3CL-1 | 69086360 | 68023642 | 10.2 | 0.01 | 98.89 | 96.34 | 45.73 |
| S3CL-2 | 71338014 | 70495282 | 10.57 | 0.01 | 98.91 | 96.43 | 45.65 |
| S3CL-3 | 61876286 | 61089042 | 9.16 | 0.01 | 98.79 | 96.04 | 45.61 |
| S3CK-1 | 56084388 | 55453120 | 8.32 | 0.01 | 98.85 | 96.21 | 45.52 |
| S3CK-2 | 71789704 | 71093070 | 10.66 | 0.01 | 98.99 | 96.64 | 45.18 |
| S3CK-3 | 70875614 | 69675430 | 10.45 | 0.01 | 98.94 | 96.5 | 45.37 |
| S4CL-1 | 63669622 | 62947798 | 9.44 | 0.01 | 99 | 96.65 | 45.42 |
| S4CL-2 | 84069564 | 82951558 | 12.44 | 0.01 | 99.01 | 96.72 | 45.38 |
| S4CL-3 | 56601852 | 55764688 | 8.36 | 0.01 | 98.86 | 96.24 | 45.55 |
| S4CK-1 | 73739032 | 72589218 | 10.89 | 0.01 | 98.88 | 96.3 | 45.56 |
| S4CK-2 | 69663020 | 68288010 | 10.24 | 0.01 | 98.92 | 96.45 | 45.22 |
| S4CK-3 | 67776526 | 66808176 | 10.02 | 0.01 | 98.96 | 96.55 | 45.34 |

S1 means at the early stage after pollination, S2 means mononuclear embryo sac stage, S3 means eight nuclear embryo sac stage, S4 means heart-shaped embryo stage. CL means apomictic treatment. CK means normal pollination treatment. 1,2,3 means repeats.


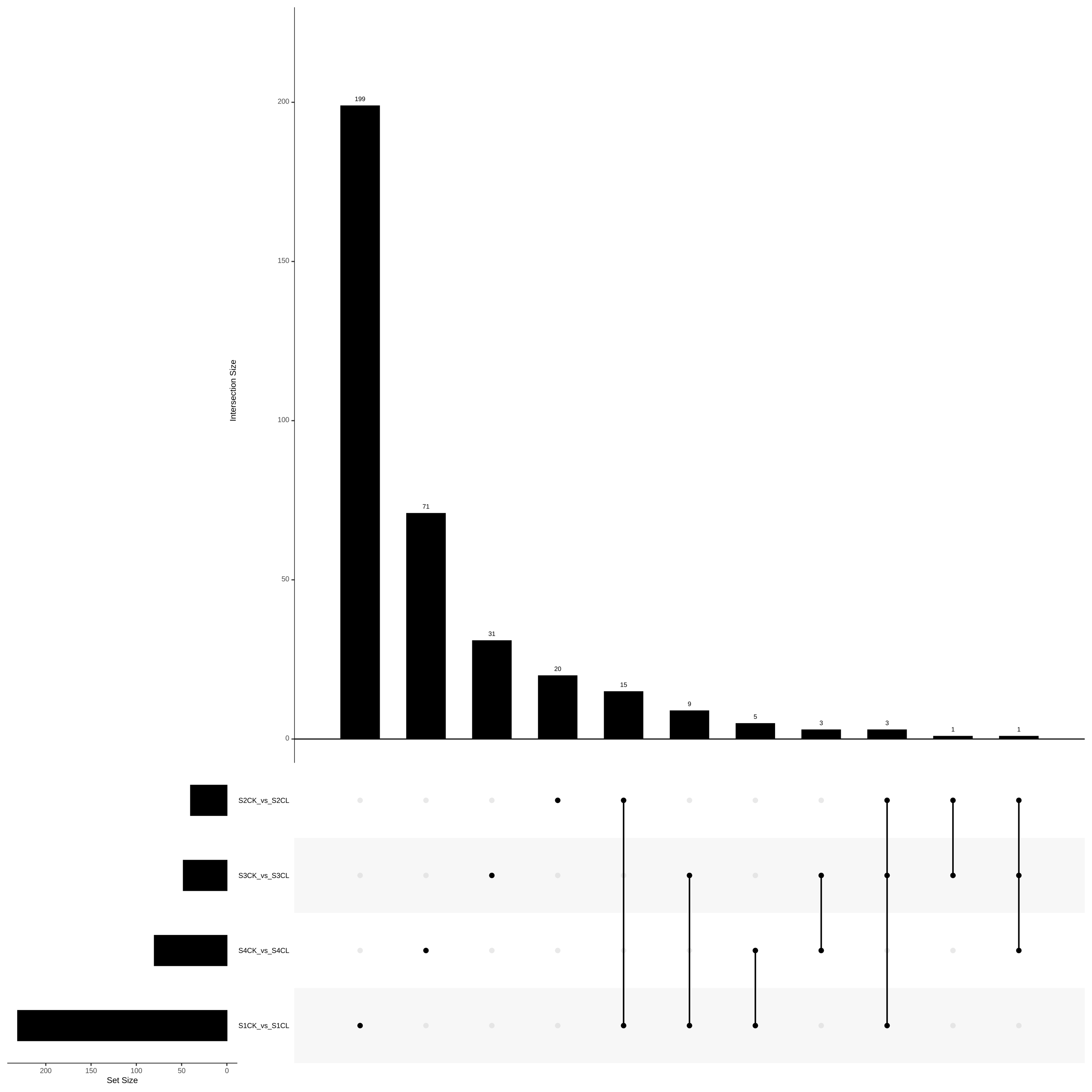


Figure S2 UpSet diagrams of DEGs

Note: The ordinate represents the amount of data of each intersection, the abscissa represents the size of the amount of data of each collection, and the black point represents the intersection of different collections represented by different filling positions.

Table S3 Results of the GO analysis of DEGs

| **Type** | **Up/Down** | **BP** | **CC** | **MF** | **Total** | **Proportion** (**%**) |
| --- | --- | --- | --- | --- | --- | --- |
|  |  | **(Biological Process)** | **(Cellular Component)** | **(Molecular Function)** |  |  |
|  | Up | 64 | 8 | 25 | 97 | 93.3 |
| GO term | Down | 44 | 7 | 14 | 65 | 62.5 |
|  | Total | 68 | 8 | 28 | 104 | 100 |
|  | Up | 977 | 230 | 399 | 1606 | 84.7 |
| Genes | Down | 159 | 51 | 80 | 290 | 15.3 |
|  | Total | 1136 | 281 | 479 | 1896 | 100 |

Table S4 GO enrichment analysis entries and pathways

| **GO terms** | **GO pathway** |
| --- | --- |
| GO:1901136 | carbohydrate derivative catabolic process |
| GO:0009611 | response to wounding |
| GO:0044550 | secondary metabolite biosynthetic process |
| GO:0000272 | polysaccharide catabolic process |
| GO:0006721 | terpenoid metabolic process |
| GO:0045892 | negative regulation of DNA-templated transcription |
| GO:0006720 | isoprenoid metabolic process |
| GO:1902679 | negative regulation of RNA biosynthetic process |
| GO:1903507 | negative regulation of nucleic acid-templated transcription |
| GO:0016114 | terpenoid biosynthetic process |
| GO:0031667 | response to nutrient levels |
| GO:0051253 | negative regulation of RNA metabolic process |
| GO:0009698 | phenylpropanoid metabolic process |
| GO:0008299 | isoprenoid biosynthetic process |
| GO:0016052 | carbohydrate catabolic process |
| GO:0045934 | negative regulation of nucleobase-containing compound metabolic process |
| GO:0048046 | apoplast |
| GO:0004497 | monooxygenase activity |
| GO:0020037 | heme binding |
| GO:0005506 | iron ion binding |
| GO:0016135 | saponin biosynthetic process |
| GO:0120251 | hydrocarbon biosynthetic process |
| GO:0016134 | saponin metabolic process |
| GO:0120252 | hydrocarbon metabolic process |
| GO:0016138 | glycoside biosynthetic process |
| GO:0016137 | glycoside metabolic process |
| GO:0046246 | terpene biosynthetic process |
| GO:0042214 | terpene metabolic process |
| GO:0002238 | response to molecule of fungal origin |
| GO:1901659 | glycosyl compound biosynthetic process |
| GO:1901657 | glycosyl compound metabolic process |
| GO:0045944 | positive regulation of transcription by RNA polymerase II |
| GO:0036294 | cellular response to decreased oxygen levels |
| GO:0071453 | cellular response to oxygen levels |
| GO:0008194 | UDP-glycosyltransferase activity |
| GO:0000978 | RNA polymerase II cis-regulatory region sequence-specific DNA binding |
| GO:0000987 | cis-regulatory region sequence-specific DNA binding |
| GO:0000977 | RNA polymerase II transcription regulatory region sequence-specific DNA binding |
| GO:0019842 | vitamin binding |
| GO:0006026 | aminoglycan catabolic process |
| GO:0006030 | chitin metabolic process |
| GO:0006032 | chitin catabolic process |
| GO:0046348 | amino sugar catabolic process |
| GO:1901072 | glucosamine-containing compound catabolic process |
| GO:1901071 | glucosamine-containing compound metabolic process |
| GO:0006022 | aminoglycan metabolic process |
| GO:0006040 | amino sugar metabolic process |
| GO:0016998 | cell wall macromolecule catabolic process |
| GO:0051259 | protein complex oligomerization |
| GO:0042542 | response to hydrogen peroxide |
| GO:0006457 | protein folding |
| GO:0009408 | response to heat |
| GO:0000302 | response to reactive oxygen species |
| GO:0051082 | unfolded protein binding |
| GO:0043621 | protein self-association |
| GO:0009723 | response to ethylene |
| GO:0006820 | anion transport |
| GO:0006073 | cellular glucan metabolic process |
| GO:0044042 | glucan metabolic process |
| GO:0009873 | ethylene-activated signaling pathway |
| GO:0071369 | cellular response to ethylene stimulus |
| GO:0000160 | phosphorelay signal transduction system |
| GO:0007623 | circadian rhythm |
| GO:0048511 | rhythmic process |
| GO:0043667 | pollen wall |
| GO:0047911 | galacturan 1,4-alpha-galacturonidase activity |
| GO:0019863 | IgE binding |
| GO:0044264 | cellular polysaccharide metabolic process |
| GO:0044877 | protein-containing complex binding |

Table S5 DEGs that might be related to apomixis

|  | |  |  | Log2FC | | | |
| --- | --- | --- | --- | --- | --- | --- | --- |
| Gene ID | Gene Index | | Gene Name | A | B | C | D |
| LOC109005849 | | primary-amine oxidase | AOC3 | 2.54 |  |  |  |
| LOC108991981 | | malate synthase | aceB | 1.31 |  |  |  |
| LOC109001928 | | caffeic acid 3-O-methyltransferase | COMT | 2.31 |  |  |  |
| LOC108987393 | | Pectinesterase | PME | 1.12 |  |  |  |
| LOC108990987 | | 3,4-dihydroxy 2-butanone 4-phosphate synthase | ribBA | 2.21 |  |  |  |
| LOC108990273 | | aromatic-L-amino-acid | TDC | 1.28 |  |  |  |
| LOC109007006 | | basic endochiti0se B | CHIB | 2.01 |  | 1.05 |  |
| LOC109012576 | | tryptophan synthase beta chain | trpB | 3.48 |  |  |  |
| LOC109020052 | | aromatic-L-amino-acid | TDC | 1.46 |  | 1.21 |  |
| LOC108993350 | | zeaxanthin epoxidase | ZEP | 1.06 |  |  |  |
| LOC118344148 | | aldehyde dehydroge0se | ALDH | 1.37 |  |  |  |
| LOC109002302 | | basic endochiti0se B | CHIB | 3.44 | 3.17 | 1.25 |  |
| LOC108996458 | | aminocyclopropanecarboxylate oxidase | ACO2 | 2.57 | 1.50 |  |  |
| LOC109012863 | | tyrosine aminotransferase | TAT | 3.09 |  |  |  |
| LOC108994833 | | glucan endo-1,3-beta-glucosidase 1/2/3 | GN1_2_3 | 2.26 |  |  |  |
| LOC108989849 | | asparagine synthase | ASNS | 1.90 |  |  |  |
| LOC108989475 | | homocysteine S-methyltransferase | BHMT2 | 2.52 |  |  |  |
| LOC108995226 | | basic endochiti0se B | CHIB | 3.02 |  |  |  |
| LOC108993352 | | zeaxanthin epoxidase | ZEP | 2.77 |  |  |  |
| LOC108986725 | | raffinose synthase | SIP2 | 2.79 |  |  |  |
| LOC109012912 | | chiti0se | CHIT | 3.01 |  | 1.76 |  |
| LOC109021870 | | branched-chain amino acid aminotransferase | ilvE | 2.29 |  |  |  |
| LOC108980942 | | beta-amylase | BAM | 1.46 |  |  |  |
| LOC109019134 | | basic endochiti0se | CHIB | 1.25 |  |  |  |
| LOC108991446 | | carbonic anhydrase | cynT | 1.43 |  |  |  |
| LOC109004165 | | chiti0se | CHIT | 1.26 |  |  | -1.28 |
| LOC108981442 | | cin0myl-alcohol dehydroge0se | MEE | 1.31 |  |  |  |
| LOC109009612 | | beta-galactosidase | GLB1 | 3.18 |  |  |  |
| LOC109021470 | | glutamine synthetase | glnA | 1.13 |  |  |  |
| LOC109013445 | | (3S,6E)-nerolidol synthase | NES1 | 8.13 |  |  |  |
| LOC108994269 | | zeaxanthin epoxidase | ZEP | 1.89 |  |  |  |
| LOC108984217 | | geranylgeranyl diphosphate synthase | GGPS | 1.48 |  |  |  |
| LOC109008126 | | 5-O-(4-coumaroyl)-D-qui0te 3'-monooxyge0se | CYP98A | 1.31 |  |  |  |
| LOC108980762 | | chiti0se | CHIT | 1.28 |  |  |  |
| LOC108990021 | | alanine-glyoxylate transami0se | AGXT2 | 1.90 |  |  |  |
| LOC108980457 | | shikimate O-hydroxycin0moyltransferase | HCT | 1.94 |  |  |  |
| LOC109014252 | | beta-glucosidase | BGLU | 1.18 |  |  |  |
| LOC109016292 | | beta-glucosidase | BGLU | 1.10 |  |  |  |
| novel.1428 | | chiti0se | CHIT | 1.06 |  |  | -1.05 |
| LOC109002304 | | basic endochiti0se B | CHIB | 1.45 |  |  |  |
| LOC108981230 | | nicotia0mine synthase | NAS |  |  | 2.32 |  |
| LOC108980790 | | chiti0se | CHIT |  |  | 2.23 |  |
| LOC108986065 | | alpha-glucosidase | malZ |  |  | 1.51 |  |
| LOC109013607 | | 1-aminocyclopropane-1-carboxylate synthase | ACS |  |  | 2.74 |  |
| LOC109009548 | | gamma-glutamylcyclotransferase | GGCT |  |  |  | -1.44 |
| LOC109006940 | | adenylyl-sulfate reductase | APR |  |  |  | -1.09 |
| LOC109012448 | | glycine hydroxymethyltransferase | glyA |  |  |  | -1.13 |
| LOC108984225 | | 3-ketoacyl-CoA synthase | KCS |  |  |  | -1.39 |
| LOC108994055 | | shikimate O-hydroxycin0moyltransferase | HCT |  |  |  | -1.70 |
| LOC108994312 | | galacturan 1,4-alpha-galacturonidase | PGA4 |  |  |  | 1.00 |
| LOC108994310 | | galacturan 1,4-alpha-galacturonidase | PGA4 |  |  |  | 1.91 |
| LOC109011192 | | glucan endo-1,3-beta-glucosidase 1/2/3 | GN1_2_3 |  |  |  | 1.62 |
| LOC108989949 | | endogluca0se | E3.2.1.4 |  |  |  | 2.89 |
| LOC109020652 | | 12-oxophytodienoic acid reductase | OPR |  |  |  | -1.11 |
| LOC108987930 | | brassinosteroid 6-oxyge0se | CYP85A1 |  |  |  | 1.24 |
| LOC108979323 | | (3S,6E)-nerolidol synthase | NES1 |  |  |  | -1.46 |
| LOC108980372 | | light-harvesting complex I chlorophyll a/b | LHCA2 |  |  |  | 1.56 |
| LOC108999828 | | riboki0se | rbsK |  |  |  | -3.46 |
| LOC118346131 | | flavonol-3-O-glucoside | FG3 | 4.37 | 3.45 |  |  |
| LOC108996228 | | flavonol-3-O-glucoside | FG3 | 5.30 |  |  |  |
| LOC108990686 | | magnesium dechelatase | SGR | 1.76 |  |  |  |
| novel.661 | | fraxetin 5-hydroxylase | CYP82C4 | 2.56 |  |  |  |
| LOC108988605 | | fraxetin 5-hydroxylase | CYP82C4 | 4.13 |  |  |  |
| LOC108987555 | | fraxetin 5-hydroxylase | CYP82C4 | 2.51 |  |  |  |
| LOC108983472 | | alpha-farnesene synthase | AFS1 | 2.05 |  |  |  |
| LOC109008521 | | Trimethyltridecatetraene | CYP82G1 | 1.10 |  |  |  |
| LOC109019039 | | flavonol-3-O-glucoside | FG3 | 2.83 | 1.63 |  |  |
| LOC108988607 | | fraxetin 5-hydroxylase | YP82C4 | 1.40 |  |  |  |
| LOC109014960 | | fraxetin 5-hydroxylase | CYP82C4 | 2.31 |  |  |  |
| LOC109012110 | | (+)-neomenthol dehydroge0se | SDR | 3.02 |  |  |  |
| LOC108995841 | | gibberellin 2beta-dioxyge0se | GA2ox | 2.66 |  |  |  |
| LOC108993447 | | gibberellin 2beta-dioxyge0se | GA2ox | 2.65 |  |  |  |
| LOC109010570 | | cytokinin dehydroge0se | CKX | 2.62 |  |  |  |
| LOC108995527 | | PHYB activation tagged suppressor 1 | CYP734A1 |  |  |  | 1.49 |
| LOC108980095 | | jasmo0te ZIM domain-containing protein | JAZ | 1.24 |  |  |  |
| LOC108998070 | | brassinosteroid insensitive 1-associated receptor kinase 1 | BAK1 | 1.64 |  |  |  |
| LOC109001963 | | jasmo0te ZIM domain-containing protein | JAZ | 1.43 |  |  |  |
| LOC108992656 | | auxin-responsive protein IAA | IAA | 1.77 |  |  |  |
| LOC108980623 | | xyloglucan:xyloglucosyl transferase TCH4 | TCH4 | 1.52 |  |  |  |
| LOC108979265 | | jasmo0te ZIM domain-containing protein | JAZ | 1.24 |  |  |  |
| LOC109009683 | | abscisic acid receptor PYR | PYL | 2.19 |  |  |  |
| LOC109007057 | | ethylene-responsive transcription factor 1 | ERF1 | 1.98 |  |  |  |
| LOC108998907 | | xyloglucosyl transferase TCH4 | TCH4 | 1.01 |  |  |  |
| LOC109008957 | | xyloglucosyl transferase TCH4 | TCH4 | 1.81 |  |  |  |
| LOC109010455 | | BRI1 ki0se inhibitor 1 | BKI1 | 1.03 |  |  |  |
| LOC109017731 | | two-component response regulator ARR-B family | ARR-B |  |  |  | 1.02 |
| LOC109009451 | | xyloglucosyl transferase TCH4 | TCH4 |  |  |  | -1.05 |
| LOC109002335 | | gibberellin receptor GID1 | GID1 |  |  |  | 1.12 |

Note: Yellow indicates up-regulation, and blue indicates down-regulation.

A: S1CL_vs_S1CK. B: S2CL_vs_S2CK. C: S3CL_vs_S3CK. D: S4CL_vs_S4CK.

S1CL_vs_S1CK means the comparison between the CL(apomictic treatment) and CK(normal pollination treatment in the stage 1 (the early stage after pollination)

S2CL_vs_S2CK means the comparison between the CL(apomictic treatment) and CK(normal pollination treatment in the stage 2 (mononuclear embryo sac stage).

S3CL_vs_S3CK means the comparison between the CL(apomictic treatment) and CK(normal pollination treatment in the stage 3 (eight nuclear embryo sac stage).

S4CL_vs_S4CK means the comparison between the CL(apomictic treatment) and CK(normal pollination treatment in the stage 4 (heart-shaped embryo stage).

A

B

| 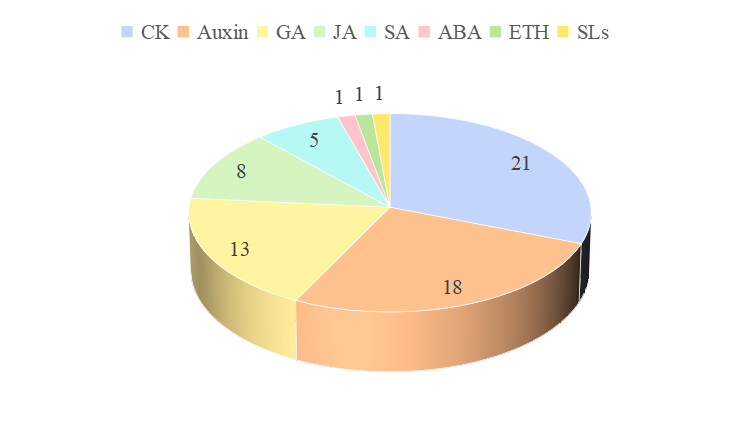 | 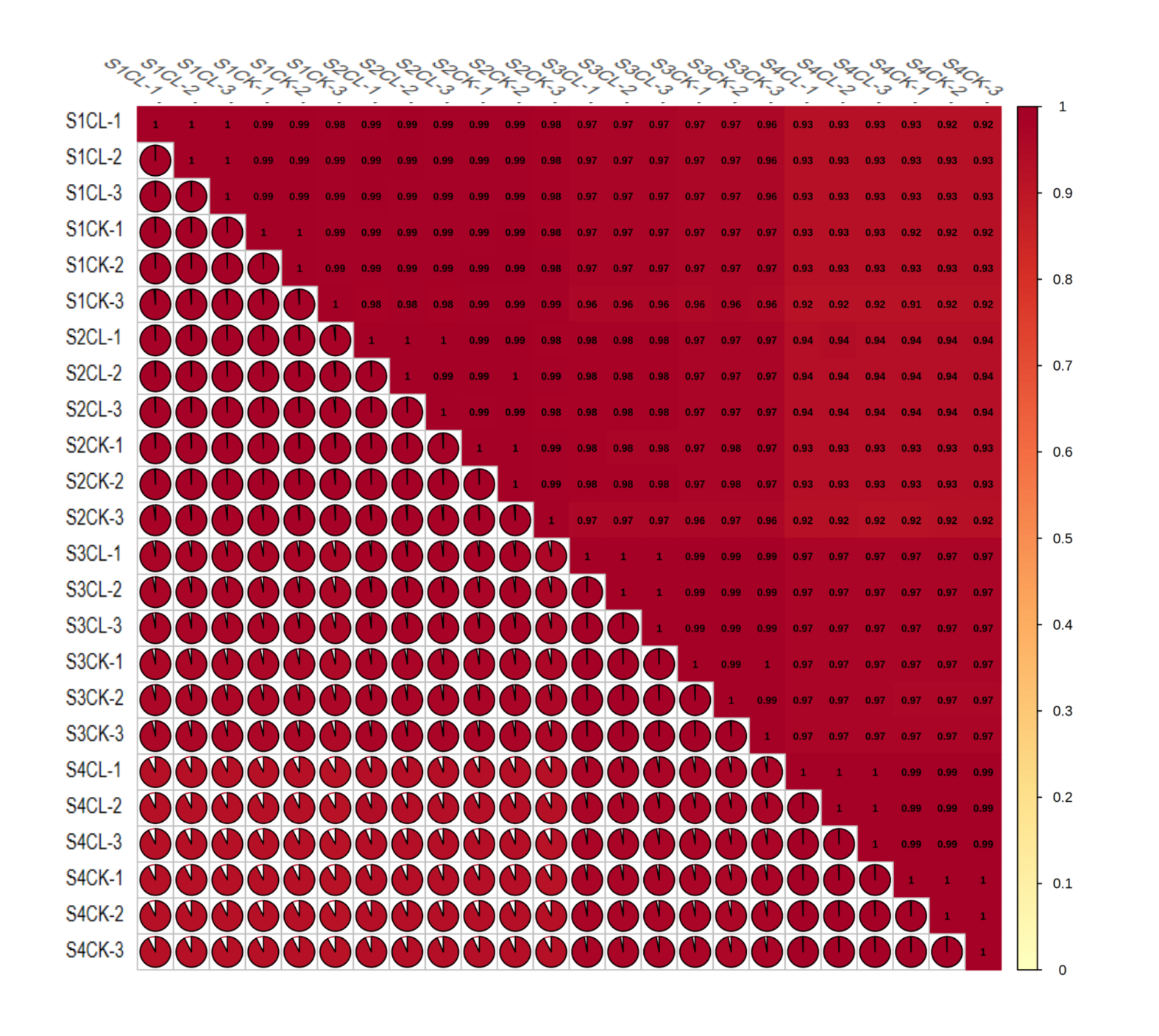 |
| --- | --- |

Figure S3 Metabolite statistics and results of the quality control analysis of mature walnut embryos

Note: A: Metabolite distribution map. Different colors represent different metabolite categories, and numbers represent the number of metabolites.

B: Sample correlation diagram. The horizontal and vertical coordinates represent different samples respectively. The difference of pie chart color represents the Pearson correlation coefficient of metabolite expression between samples. The red the color means the stronger the positive correlation between metabolite expression of samples. The yellower the color means the weaker the correlation between metabolite expression of samples.

S1 means at the early stage after pollination, S2 means mononuclear embryo sac stage, S3 means eight nuclear embryo sac stage, S4 means heart-shaped embryo stage. CL means apomictic treatment. CK means normal pollination treatment. 1,2,3 means repeats.

Table S6 The result of DAMs analysis between the CL and CK

| **Substance** | **Abbreviations** | **Category** | **Type** |
| --- | --- | --- | --- |
| 3-Indoleacetonitrile | IAN | Auxin | up |
| Tryptamine | TRA | Auxin | up |
| Indole-3-acetyl-L-valine methyl ester | IAA-Val-Me | Auxin | down |
| Methyl indole-3-acetate | MEIAA | Auxin | up |
| meta-Topolin | mT | CK | up |
| Kinetin-9-glucoside | K9G | CK | down |
| 6-Benzyladenine | BAP | CK | up |
| Gibberellin A51 | GA51 | GA | up |
| Gibberellin A5 | GA5 | GA | up |
| Gibberellin A29 | GA29 | GA | up |
| cis(+)-12-Oxophytodienoic acid | OPDA | JA | down |
| Jasmonic acid | JA | JA | up |
| N-[(-)-Jasmonoyl]-(L)-valine | JA-Val | JA | up |
| Methyl jasmonate | MEJA | JA | down |
| N-[(-)-Jasmonoyl]-(l)-phenylalanine | JA-Phe | JA | up |
| Jasmonoyl-L-isoleucine | JA-ILE | JA | up |
| 2-Methoxycarbonylphenyl beta-D-glucopyranoside | MeSAG | SA | up |
| 2-Coumarate | 2-Coumarate | SA | up |
| (±) Strigol | ST | SL | down |

Table S7 Gene annotation information

| Gene name | Gene ID | Gene name | Gene ID | Gene name | Gene ID |
| --- | --- | --- | --- | --- | --- |
| *BAK1* | LOC108998070 | *AOC3/tynA* | LOC109005849 | *CYP82G1* | LOC109008521 |
| *COMT* | LOC109001928 | *asnB/ASN* | LOC108989849 | *aceB/glcB* | LOC108991981 |
| *ALDH* | LOC118344148 | *SGR* | LOC108990686 | *SDR* | LOC109012110 |
| *trpB* | LOC109012576 | *IAA* | LOC108992656 | *ribBA* | LOC108990987 |
| *ACO* | LOC108996458 | *PME* | LOC108987393 | *AFS1* | LOC108983472 |
| *GN1_2_3* | LOC108994833 | *TAT* | LOC109012863 | *BHMT2* | LOC108989475 |
| *JAZ* | LOC108980095  LOC109001963 | *DDC/TDC* | LOC108990273 LOC109020052 | *ZEP/ABA1* | LOC108993352 LOC108993350 |
| *FG3* | LOC109019039 LOC118346131 LOC108996228 | *CHIB* | LOC109002302 LOC108995226 LOC109007006 | *CYP82C4* | novel.661  LOC108987555 LOC108988605 LOC109014960 LOC108988607 |

Table S8 The expression of differential metabolites in different walnut embryos

| **Index** | **The expression of CL** | **The expression of CK** | **Multiple** |
| --- | --- | --- | --- |
| TRA | 14541.65 | 4831.44 | 3.01 |
| JA | 32.44 | 9.38 | 3.46 |
| JA-ILE | 11.6 | 1.29 | 8.97 |

| 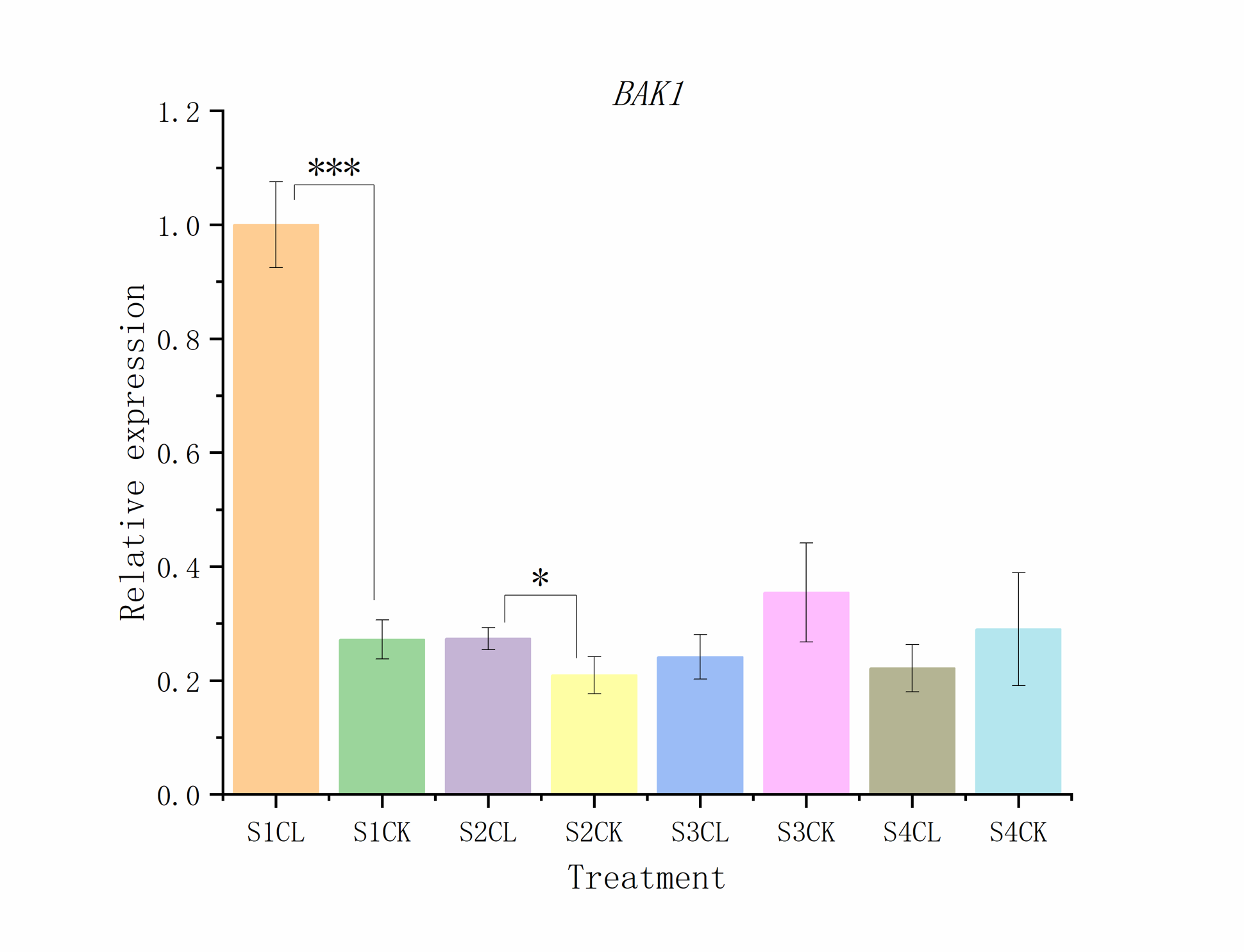 | 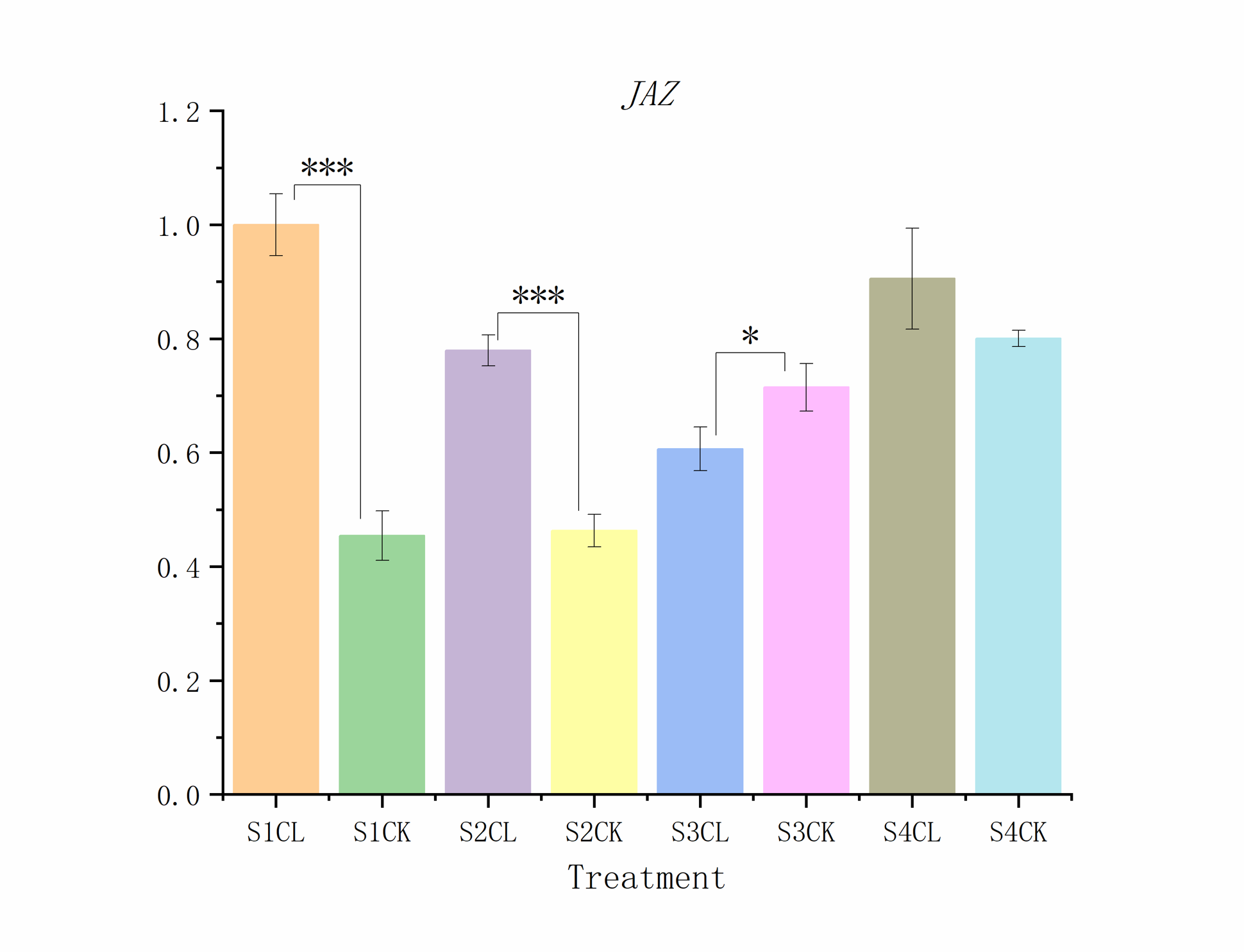 | 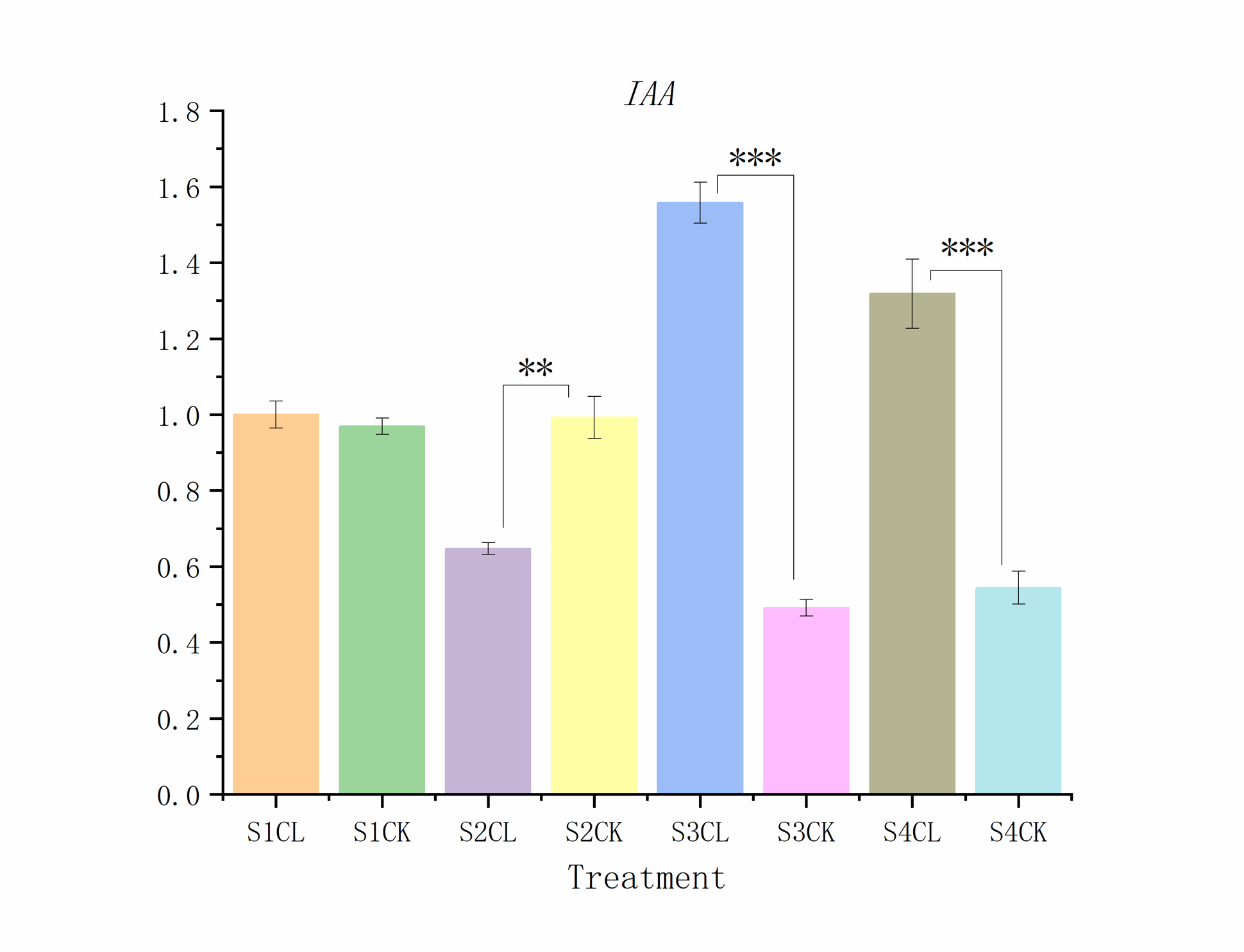 |
| --- | --- | --- |
| 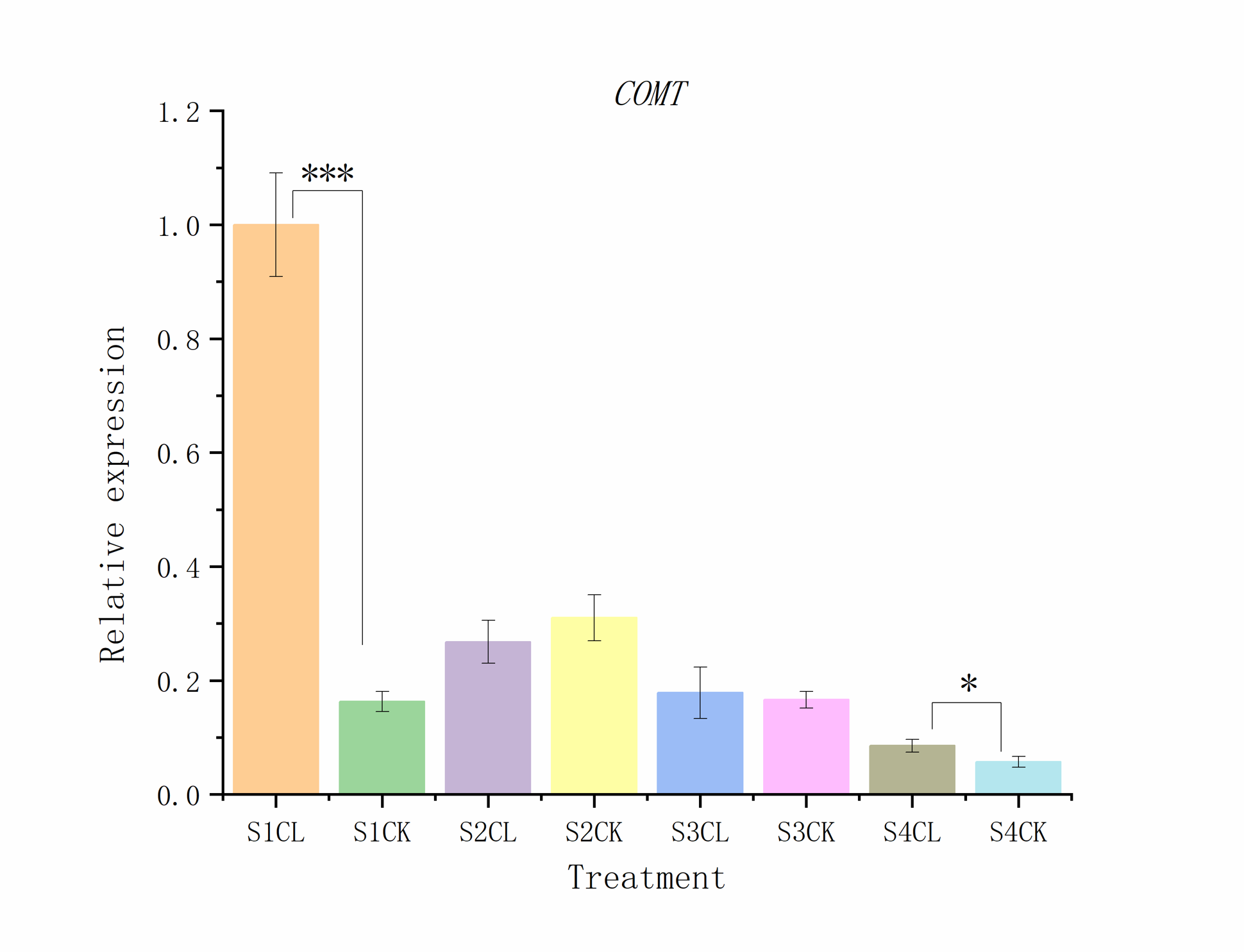 | 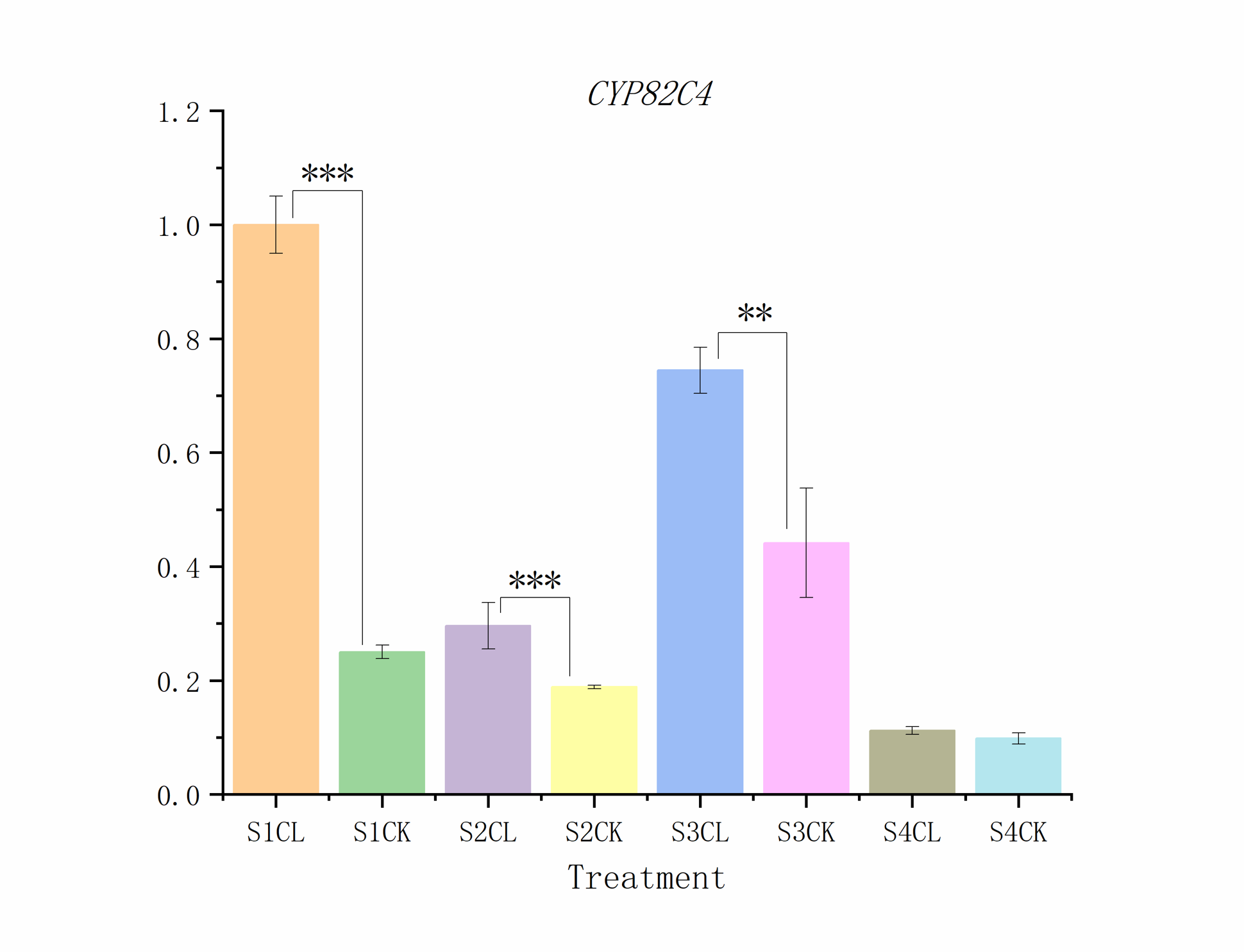 | 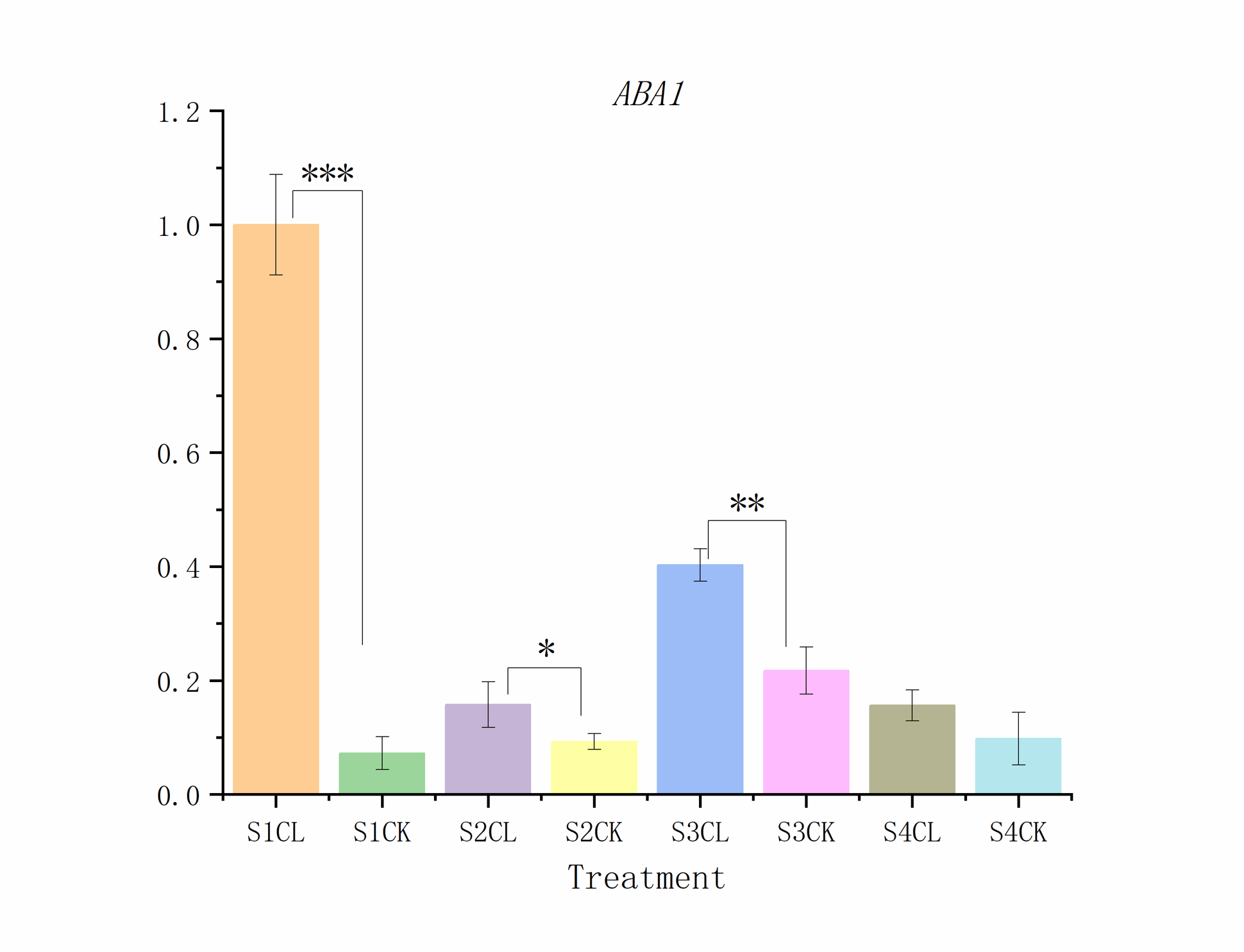 |
| 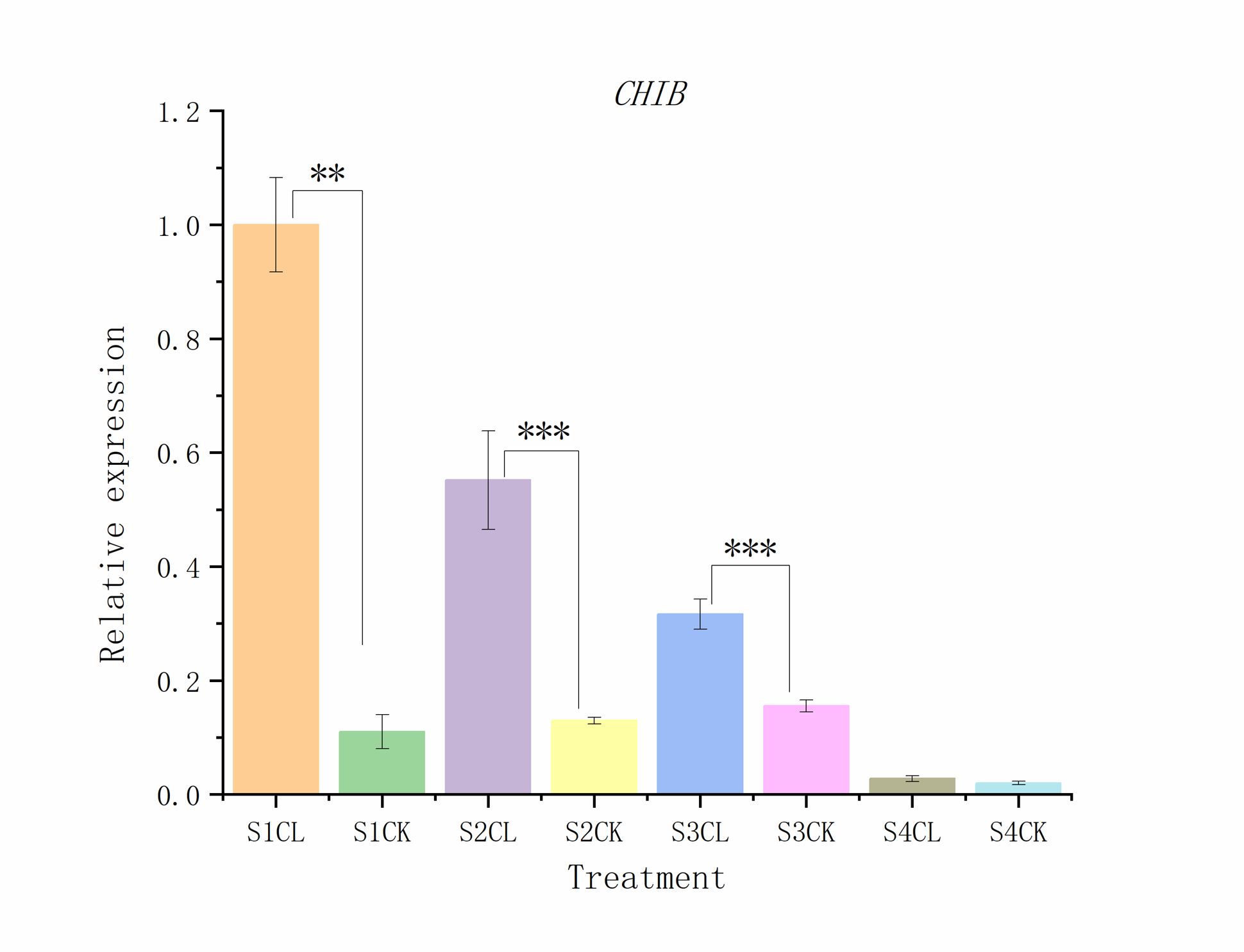 | 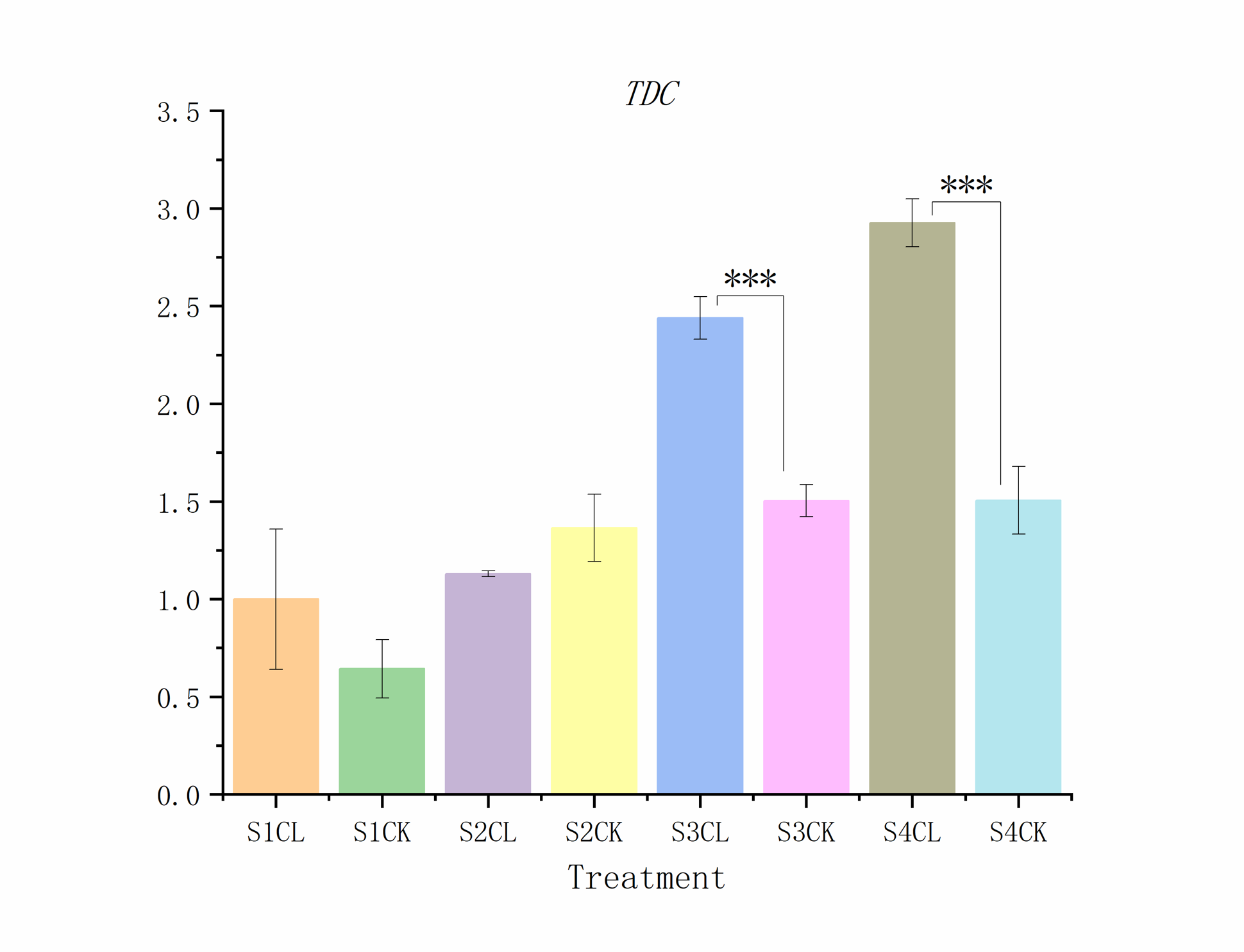 | 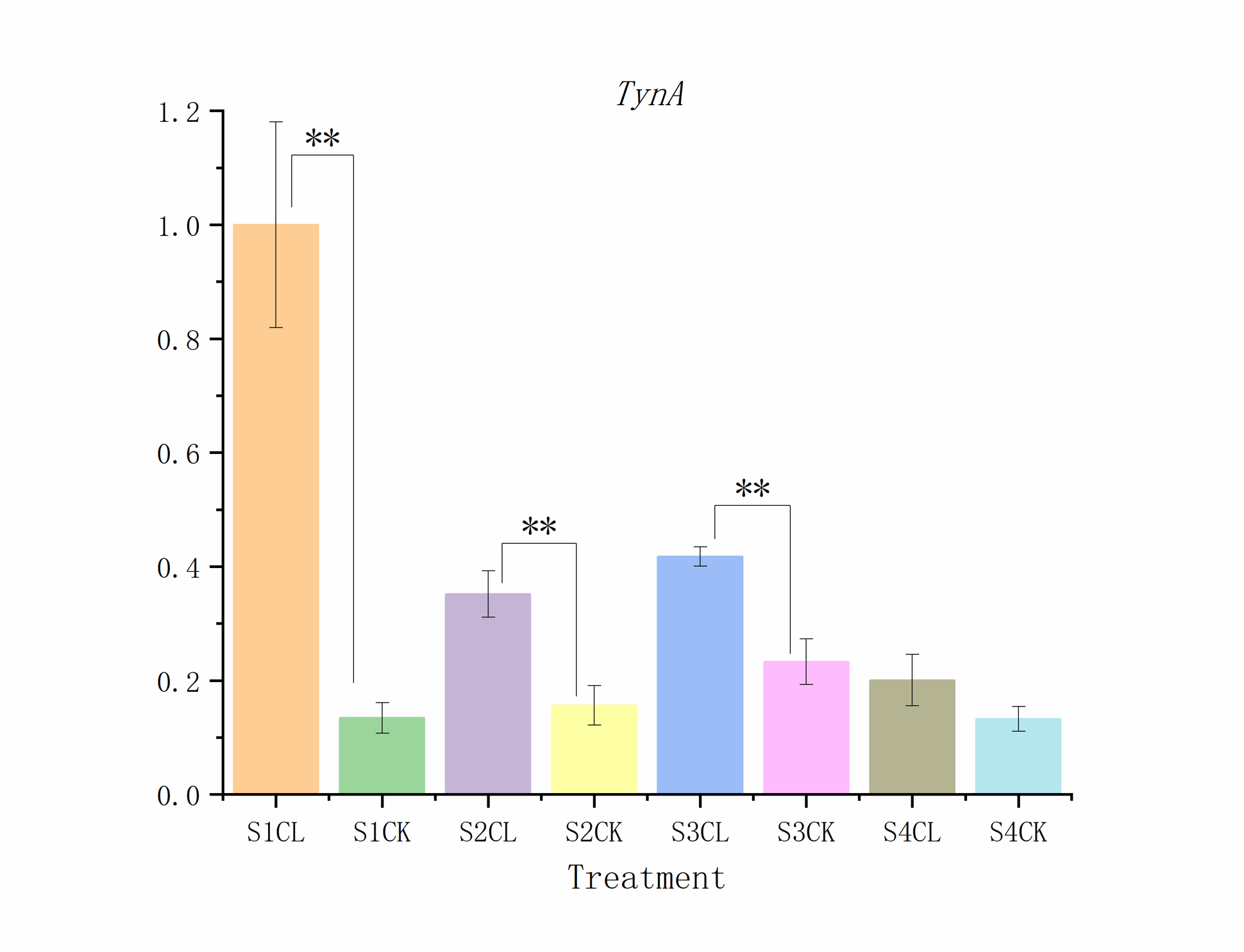 |

Figure S4 qRT-PCR validation results

Note: The ordinate represents the relative expression, and the abscissa represents the processing. Different colors represent different treatments. *, * *, and * * * indicate significant or extremely significant differences at *P* < 0.05, *P* < 0.01, and *P* < 0.001 levels, respectively.

*BAK1*: brassinosteroid insensitive 1-associated receptor kinase 1. *JAZ*: jasmonate ZIM domain-containing protein. *IAA*: auxin-responsive protein *IAA*. *CYP82C4*: fraxetin 5-hydroxylase. *COMT*: caffeic acid 3-O-methyltransferase. *ABA1*: zeaxanthin epoxidase. *CHIB*: basic endochitinase B. *TDC*: L-tryptophan decarboxylase. *TynA*: primary-amine oxidase.
